# DNA barcoding evaluation and implications for phylogenetic relationships in Lauraceae from China

**DOI:** 10.1371/journal.pone.0175788

**Published:** 2017-04-17

**Authors:** Zhi-Fang Liu, Xiu-Qin Ci, Lang Li, Hsi-Wen Li, John G. Conran, Jie Li

**Affiliations:** 1 Plant Phylogenetics and Conservation Group, Center for Integrative Conservation, Xishuangbanna Tropical Botanical Garden, Chinese Academy of Sciences, Kunming, Yunnan, P. R. China; 2 University of Chinese Academy of Sciences, Beijing, P. R. China; 3 Herbarium (KUN), Kunming Institute of Botany, Chinese Academy of Sciences, Kunming, Yunnan, P. R. China; 4 Australian Centre for Evolutionary Biology and Biodiversity & Sprigg Geobiology Centre, School of Biological Sciences, University of Adelaide, Adelaide, SA, Australia; Institute of Botany, CHINA

## Abstract

Lauraceae are an important component of tropical and subtropical forests and have major ecological and economic significance. Owing to lack of clear-cut morphological differences between genera and species, this family is an ideal case for testing the efficacy of DNA barcoding in the identification and discrimination of species and genera. In this study, we evaluated five widely recommended plant DNA barcode loci *mat*K, *rbc*L, *trn*H–*psb*A, ITS2 and the entire ITS region for 409 individuals representing 133 species, 12 genera from China. We tested the ability of DNA barcoding to distinguish species and as an alternative tool for correcting species misidentification. We also used the *rbc*L+*mat*K+*trn*H–*psb*A+ITS loci to investigate the phylogenetic relationships of the species examined. Among the gene regions and their combinations, ITS was the most efficient for identifying species (57.5%) and genera (70%). DNA barcoding also had a positive role for correcting species misidentification (10.8%). Furthermore, based on the results of the phylogenetic analyses, Chinese Lauraceae species formed three supported monophyletic clades, with the *Cryptocarya* group strongly supported (PP = 1.00, BS = 100%) and the clade including the *Persea* group, Laureae and *Cinnamomum* also receiving strong support (PP = 1.00, BS = 98%), whereas the *Caryodaphnopsis*–*Neocinnamomum* received only moderate support (PP = 1.00 and BS = 85%). This study indicates that molecular barcoding can assist in screening difficult to identify families like Lauraceae, detecting errors of species identification, as well as helping to reconstruct phylogenetic relationships. DNA barcoding can thus help with large-scale biodiversity inventories and rare species conservation by improving accuracy, as well as reducing time and costs associated with species identification.

## Introduction

Lauraceae in China comprise about 25 widely distributed genera (two endemic, two introduced) with 445 species (316 endemic, three introduced) [1, 2]. The family has major ecological and economic importance [3]. They play an important role in tropical and subtropical forests, often as canopy dominants [1, 4, 5–7] and also have economic significance as sources of medicine, timber, spices, nutritious fruits and perfumes [1, 8, 9]. The fruits of some species contain abundant oil and fat [1]. However, because of their biological and economic relevance, some plants have been overexploitated [10], with 109 species now listed as endangered by the China Species Red List [10].

Because it is important to know whether economic benefit estimates are stable over time [11], the accurate identification of species is important for their protection. However, the taxonomy of Lauraceae, as with other taxonomically complex groups of angiosperms (*e*.*g*., *Berberis*: Roy *et al*. [12]; *Ficus*: Li *et al*. [13]; *Curcuma*: Chen *et al*. [14]; *Salix*: Percy *et al*. [15]; *Rhododendron*: Yan *et al*. [16]), is very poorly resolved. The classification traditionally has been based mainly on morphological characters, such as phyllotaxis, perianth, inflorescence type, size of tepals, number of fertile stamens, number of locules per anther, or fate of tepals in fruit [17–20]. Chinese Lauraceae represent more than 400 species and encompass a broad range of morphological diversity [1], both reproductive and vegetative [21], of which the former are regarded as more effective for classification and identification. However, as most species are tall trees with small, inconspicuous flowers that are not easy to locate or collect in the field [3], generic delimitation within the family is problematic [17–20], particularly as many trees are sterile when sampled (expecially during biodiversity inventories) and must be identified using vegetative characters [21], making misidentifications inevitable [22]. Even with flowers and fruits, the identification and discrimination of Lauraceae taxa can be challenging for non-specialists [4, 21, 23]. So far, scant information is available regarding accurate classification and biodiversity assessment within this family, particularly in south-east Asia, so complementary methods of identification and classification are urgently needed for Lauraceae.

DNA barcoding is a technique for taxonomic identification using one or several standardized DNA regions that are universally present in the target lineages and have sufficient sequence variation to recognize species and identify individuals correctly [24–29]. In order to choose universal DNA barcodes for plants, various molecular markers have been analyzed, including the cpDNA regions, *mat*K, *rbc*L and *trn*H–*psb*A [27, 28, 30, 31], as well as nuclear DNA regions such as ITS (ITS1+5.8S+ITS2) and ITS2 only [32–35]. These regions were chosen based on three main criteria: (a) universality, (b) sequence quality and (c) discriminatory power [29]. DNA barcoding has been shown to be an important tool for species identification and as a supplement to traditional morphology-based taxonomy [36, 37, 38]. Nevertheless, relatively little attention has been paid to sources of potential bias which affect species identification error rates [39], even though species-level barcodes can be prone to substantial errors related to morphological identifications [21]. Combining DNA sequences with existing morphological characters may facilitate species identification and classification [38, 40, 41], as well as broaden our understanding of phylogenetic signal within target lineages [42]. Previous studies have shown that it is not easy to reconstruct phylogenetic relationships in Lauraceae [7, 41, 43–46], so the affinities of species and generic relationships within many of the major lineages in the family are still poorly resolved [7, 18, 43, 44, 47, 48].

Accordingly, in the present study we used existing molecular barcodes: three cpDNA regions (*rbc*L, *mat*K, *psb*A–*trn*H) and the nuclear marker ITS (ITS1+5.8S+ITS2), as well as the subunit ITS2, to examine the taxonomic classification and phylogeny of Lauraceae. Our main aims were to:

1. evaluate barcode universality in Chinese Lauraceae species;
2. assess DNA barcoding performance relative to species identification;
3. determine if these barcodes can also allow for the reconstruction of phylogenetic relationships within the Lauraceae, relative to previously recognized subdivisions and affinities.

## Materials and methods

### Ethics statement

Collection of these species was conducted in compliance with existing regulations for plants defined as non-commercial, as determined by local government offices. In addition, these sample collections were performed in China with the written approval from the National Forest Bureau and relevant local governments, complying with Chinese and international regulations for the collection of native plant samples.

### Sampling

A total of 409 individuals of 133 species from 12 genera of Lauraceae were included in this study (S1 and S2 Tables, Supporting Information), distributed across eight provinces: Chongqing, Guangdong, Guangxi, Hainan, Hunan, Sichuan, Yunnan and Zhejiang, representing much of the diversity of this family in China. Materials for this study were collected in the field from 2002 to 2012, with 22 species represented by a single individual and 111 species represented by two to nine individuals (an average of three samples per species). The Lauraceae expert at KUN, Hsi-Wen Li, who is one of the co-authors, identified the vouchers (S1 Table) based on the reproductive or vegetative characters available. All vouchers were stored at the Herbarium of Xishuangbanna Tropical Botanical Garden (HITBC).

### DNA isolation, amplification and sequencing

Total genomic DNA was extracted from silica gel-dried leaf tissue or herbarium specimens using a modified CTAB method [49]. The plastid markers *rbc*L, *mat*K, *trn*H–*psb*A and nuclear markers ITS and ITS2 were amplified using multiple primers, following the suggestions of Dunning and Savolainen [50] and Yu *et al*. [51]. For example, four primers sets were tested for *mat*K due to its generally poor performance of amplification and sequencing [52]. DMSO and BSA were also added to enhance the PCR performance for *mat*K and ITS [53, 54].

PCR was performed in 20 μL reaction mixtures containing 0.2 μL of Taq polymerase (5 U), 2.0 μL of 10 × PCR buffer, 2.0 μL of 25 mM MgCl_2_, 2 μL of 2.5 mM dNTPs, 1 μL of 10 uM of each primer, 1 μL of DMSO, 2 μL of 1 mg/ml BSA and 2 μL template DNA. For primer combinations, PCR thermal conditions and references, see Supporting Information (S3 Table). All PCR products were sequenced at the Beijing Genomics Institute (BGI).

### Sequence editing and alignment

Raw sequences were assembled and edited using Sequencher 4.14 (GeneCodes Corp., Ann Arbor, Michigan, USA) and deposited in GenBank (see S2 Table for GenBank accession numbers). Edited sequences were then aligned using Geneious 6.1.2 (Biomatters Ltd.), Clustal W [55] and MUSCLE [56], with final manual adjustment undertaken with Geneious 6.1.2 and BioEdit 7.0.9.0 [57]. All variable sites were rechecked on the original trace files for final confirmation. For the *rbc*L and *mat*K markers, a global multiple sequence alignment was used. The *rbc*L sequences were unambiguous, due to the absence of insertions or deletions, but alignment of *mat*K was more difficult due to the insertion of triplet codons, so the alignment results were checked visually. The *trn*H–*psb*A and ITS sequences were highly variable and very difficult to align with Geneious, so these markers were aligned several times by Clustal W and MUSCLE and then a supermatrix was created by concatenating them with the aligned sequences of the remaining markers.

### Data analysis

Two widely applied methods (tree-based and similarity-based) were used to evaluate species discrimination success, following Huang *et al*. [22]. Five single markers and all possible combinations were applied. For the tree-based method, we used Geneious 6.1.2 to construct Neighbour-Joining (NJ) trees. For the similarity-based method, we used BLAST [58] for building local reference databases against which all sequences were then queried using the blastn program. The 22 species with only a single individual were excluded in NJ trees and BLAST (n ≥ 2) analyses. Species discrimination was considered successful only when all conspecific individuals formed a single clade supported by bootstrap values greater than 50% in the NJ tree [59], and when all individuals of the species or genus only had a top matching hit with a conspecific/congeneric individual in BLAST (the query sequence itself was excluded from the list of top hits when there were multiple individuals).

**Fig 1:**
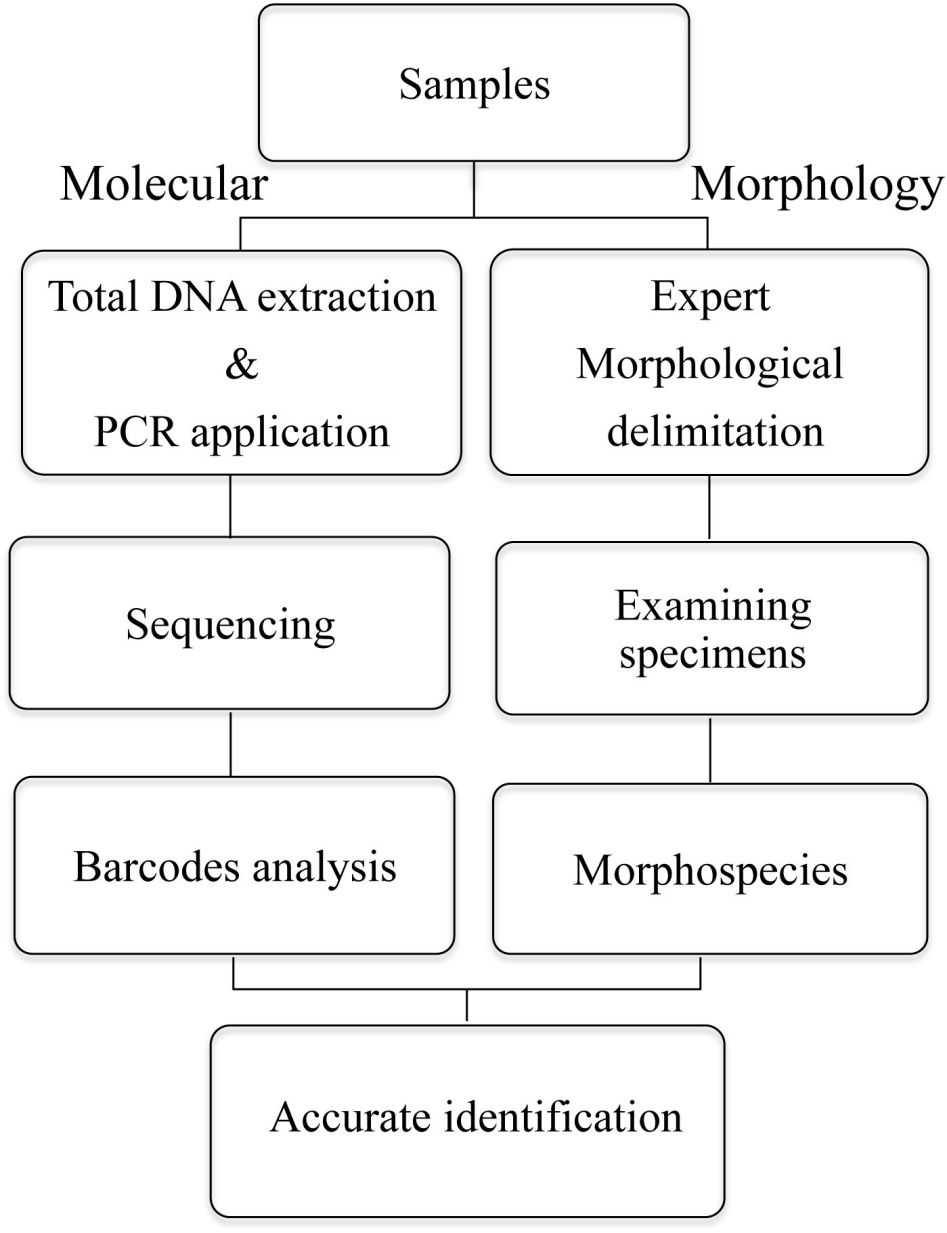
A schematic pipelines of conventional and molecular species identification analyses.

In detecting identification errors, a two-step procedure of reciprocal illumination was used. We evaluated errors in the initial morphology-based identifications combining morphology and DNA sequence data to uncover and correct mistakes in Lauraceae identification. A schematic illustration is used to show the identification process in the present study (Fig 1). Firstly, our initial morphological delimitations were identified by the Lauraceae expert and defined as morphospecies. Then we compared the specimens with herbarium specimens from HITBC, KUN and PYU. Finally, we combined DNA sequences with existing morphological characters. Potential errors were identified through examination of the NJ trees (using *rbc*L, *mat*K and the combination of *rbc*L+*mat*K+*trn*H–*psb*A+ITS) and BLAST. If the result indicated that the sample did not belong to an *a priori* assigned taxon, it was flagged as a possible error and the sample was then compared with descriptions and herbarium specimens of the species involved, using morphological characteristics in order to confirm whether an error had been made.

In phylogenetic analyses, combined data sets are often able to generate more resolved and better-supported phylogenies [41, 60], so this approach was also used for Lauraceae. In this study, phylogenetic analyses are inferred from sequence variation in the four-locus combination of *rbc*L+*mat*K+*trn*H–*psb*A+ITS. Bayesian Inference (BI) and Maximum Parsimony (MP) phylogenetic analyses were conducted to reconstruct phylogenetic relationships using PAUP_*_4.0b10 [61] and MrBayes 3.1.2 [62], with gaps coded as simple indels using the program Gapcoder [63]. For the Bayesian analysis, the dataset was partitioned by markers. Modeltest 3.7 [64, 65] was used to select the best-fit evolutionary model for each partition according to the Akaike Information Criterion (AIC) [66]. The Markov chain Monte Carlo (MCMC) algorithm was run with one cold and three heated chains for 5,000,000 generations, which started from random trees and sampling one out of every 500 generations. Inspection of the log likelihood values suggested that stationarity was reached well before the first 25% implemented as default value for the burn-in and the remaining 75% were used for constructing the consensus tree with the proportion of bifurcations found in this consensus tree given as posterior probabilities (PP). MP analysis was conducted using the following heuristic search options: tree-bisection-reconnection (TBR) branch swapping, collapse of zero length branches and MulTrees on, with 1000 random taxon additions, saving 100 trees from each random sequence addition [66]. All character states were regarded as unordered and equally weighted. Bootstrap support values (BS) for internal nodes were estimated with 100 heuristic bootstrap replicates. The reliability of clades as judged by the posterior probability in Bayesian analysis was generally higher than that as judged by the bootstrap probability in MP analysis [67]. Based on known phylogenies and simulations, bootstrap values of 50% corresponding to posterior probabilities of 90% are generally considered as moderate support of true clade probabilities, and a strong relationship between bootstrap values of 70% corresponding to posterior probabilities of 95% are generally considered as strong support [68, 69]. Three species of Monimiaceae, plus *Gomortega nitida* Ruiz & Pav. (Gomortegaceae) were selected as outgroups, based on their sister relationship to Lauraceae in a previous study [7]. A sample of the monotypic African genus *Hypodaphnis* was also included, as the genus is considered to be sister to the remainder of Lauraceae [7], with ITS sequences for these five species downloaded from GenBank.

## Results

### Barcode universality and sequence characteristics

In total, we obtained 1474 sequences from the 409 samples, representing 133 species of 12 genera after correction. These included 381 sequences for *rbc*L, 381 sequences for *mat*K, 323 for *trn*H–*psb*A, 228 for ITS2 and 161 for ITS (S2 Table). Sequence recovery success rates were very high for *rbc*L (92.5% of individuals, 97.7% of species, 100% of genera) and *mat*K (92.5% of individuals, 92.5% of species, 100% of genera) with the four commonly used primers pairs *mat*K-1RKIM-f/*mat*K-3FKIM-r, *mat*K-472f/*mat*K-1248r, *mat*K-390f/*mat*K-1326r and *mat*K-xf/*mat*K-5r. Two regions showed moderate success: *trn*H–*psb*A region (78.4% of individuals, 86.5% of species, 100% of genera) and ITS2 (55.3% of individuals, 73.7% of species, 100% of genera). In contrast, the ITS region showed the lowest overall recovery rates (39.1% of individuals, 57.9% of species, 100% of genera) (see Table 1).

**Table 1:** Sequence recovery rates for five DNA barcodes evaluated in this study.

Barcode regions	*rbc*L	*mat*K	*trn*H-*psb*A	ITS	ITS2
Successful individuals/sampled individuals	381/412 (92.5%)	381/412 (92.5%)	323/412 (78.4%)	161/412 (39.1%)	228/412 (55.3%)
Successful species/sampled species	130/133 (97.7%)	123/133 (92.5%)	115/133 (86.5%)	77/133 (57.9%)	98/133 (73.7%)
Successful genera/sampled genera	12/12 (100%)	12/12 (100%)	12/12 (100%)	12/12 (100%)	12/12 (100%)

For each category, the absolute number of successes is given along with the percentage relative to the total number. Successful individuals/sampled individuals; Successful species/sampled species; Successful genera/sampled genera (n ≥ 1).

### Mistakes in taxonomic identification

**Fig 2:**
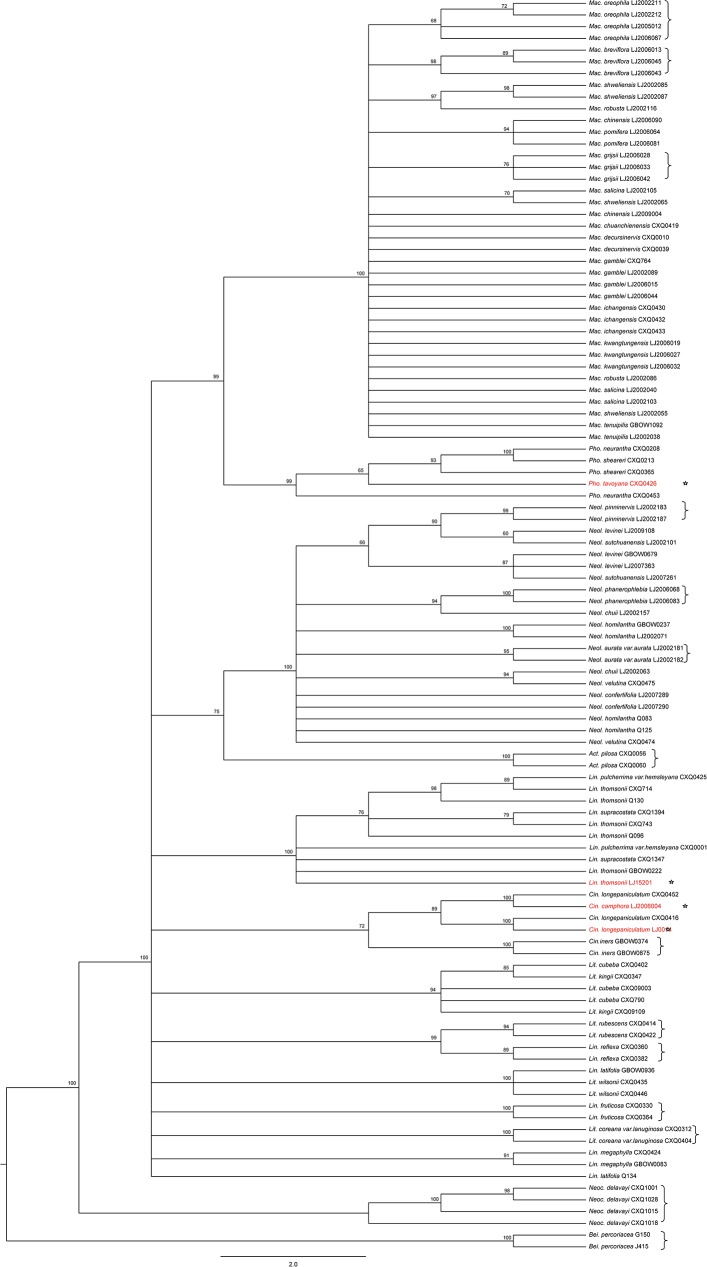
Species misidentification and resolution at the genus and species levels. The NJ tree based on the combined barcodes *rbc*L+*mat*K+*trn*H–*psb*A+ITS. The bootstrap values ≥ 50% are shown on the branches. The stars represent corrected individuals; brackets represent successfully identified species.

After combining DNA sequences with existing morphological characters, various putative species were found to comprise 1–4 individuals that were divergent from the majority of individuals sequenced for their species and that were nested within other species. In these cases, a detailed reanalysis of voucher specimens combined with NJ Tree analyses and BLAST examinations was needed. The results showed that the divergent individuals had been identified incorrectly. In total, 44 individuals (10.8%) had been misidentified by the expert (Table 2, Fig 2; S1 and S2 Figs), 34 at the generic level and 10 at the species level. Following these corrections, we recognised 133 OTUs for the study. The misidentified samples and their identification after revision are listed in Table 2.

**Table 2:** Original species determinations and correct species using DNA barcodes.

Original species	Correct species
*Alseodaphne andersonii J116*	*Cryptocarya acutifolia J116*
*Alseodaphne andersonii J127*	*Cryptocarya acutifolia J127*
*Alseodaphne petiolaris J470*	*Cryptocarya acutifolia J470*
*Beilschmiedia robusta G174*	*Beilschmiedia purpurascens G174*
*Beilschmiedia yunnanensis J467*	*Beilschmiedia purpurascens J467*
*Beilschmiedia yunnanensis GBOW0246*	*Machilus robusta GBOW0246*
*Beilschmiedia yunnanensis GBOW0678*	*Machilus yunnanensis GBOW0678*
*Cinnamomum chartophyllum J088*	*Beilschmiedia yunnanensis J088*
*Cinnamomum chartophyllum J193*	*Beilschmiedia yunnanensis J193*
*Cinnamomum mollifolium J677*	*Beilschmiedia roxburghiana J677*
*Cinnamomum tenuipilum J083*	*Litsea acutivena J083*
*Cryptocarya brachythyrsa J576*	*Beilschmiedia brachythyrsa J576*
*Cryptocarya calcicola L061*	*Beilschmiedia purpurascens L061*
*Cryptocarya calcicola J607*	*Cryptocarya acutifolia J607*
*Cryptocarya chinensis J386*	*Beilschmiedia yunnanensis J386*
*Cryptocarya chinensis J407*	*Litsea lancilimba J407*
*Cryptocarya densiflora GBOW0745*	*Caryodaphnopsis laotica GBOW0745*
*Cryptocarya yunnanensis J485*	*Beilschmiedia yunnanensis J485*
*Lindera latifolia CXQ09023*	*Phoebe neurantha CXQ09023*
*Lindera latifolia GBOW0930*	*Machilus grijsii GBOW0930*
*Lindera latifolia GBOW0936*	*Machilus grijsii GBOW0936*
*Litsea baviensis J227*	*Litsea pierrei J227*
*Litsea elongata G102*	*Litsea salicifolia G102*
*Litsea elongata G198*	*Litsea salicifolia G198*
*Litsea elongata J092*	*Litsea acutivena J092*
*Litsea euosma LJ2002068*	*Neolitsea chuii LJ2002068*
*Litsea glutinosa J133*	*Actinodaphne henryi J133*
*Litsea rotundifolia CXQ0069*	*Cryptocary calcicola CXQ0069*
*Machilus chuanchienensis CXQ0426*	*Phoebe tavoyana CXQ0426*
*Machilus pauhoi CXQ0080*	*Litsea greenmaniana* var. *angustifolia CXQ0080*
*Machilus salicina Q133*	*Litsea greenmaniana* var. *angustifolia Q133*
*Machilus viridis Q090*	*Cinnamomum chago Q090*
*Machilus viridis Q129*	*Cinnamomum chago Q129*
*Machilus viridis CXQ690*	*Cinnamomum chago CXQ690*
*Machilus viridis CXQ762*	*Cinnamomum chago CXQ762*
*Machilus yunnanensis LJ2002064*	*Cinnamomum chago LJ2002064*
*Machilus yunnanensis LJ2002072*	*Cinnamomum chago LJ2002072*
*Neolitsea levinei LJ2002035*	*Machilus tenuipilis LJ2002035*
*Neolitsea lunglingensis LJ0014*	*Cinnamomum longepaniculatum LJ0014*
*Neolitsea lunglingensis LJ15201*	*Lindera thomsonii LJ15201*
*Neolitsea lunglingensis LJ2002058*	*Neolitsea homilantha LJ2002058*
*Neolitsea phanerophlebia LJ2006004*	*Cinnamomum camphora LJ2006004*
*Neolitsea phanerophlebia LJ2006083*	*Neolitsea chuii LJ2006083*
*Neolitsea shingningensis CXQ0284*	*Lindera fragrans CXQ0284*

Original species determinations based on morphological characters; correct species based on NJ trees of *mat*K, *rbc*L and *rbc*L+*mat*K+*trn*H–*psb*A+ITS and BLAST plus re-examination of morphology.

### Discrimination efficiency in Lauraceae

**Fig 3:**
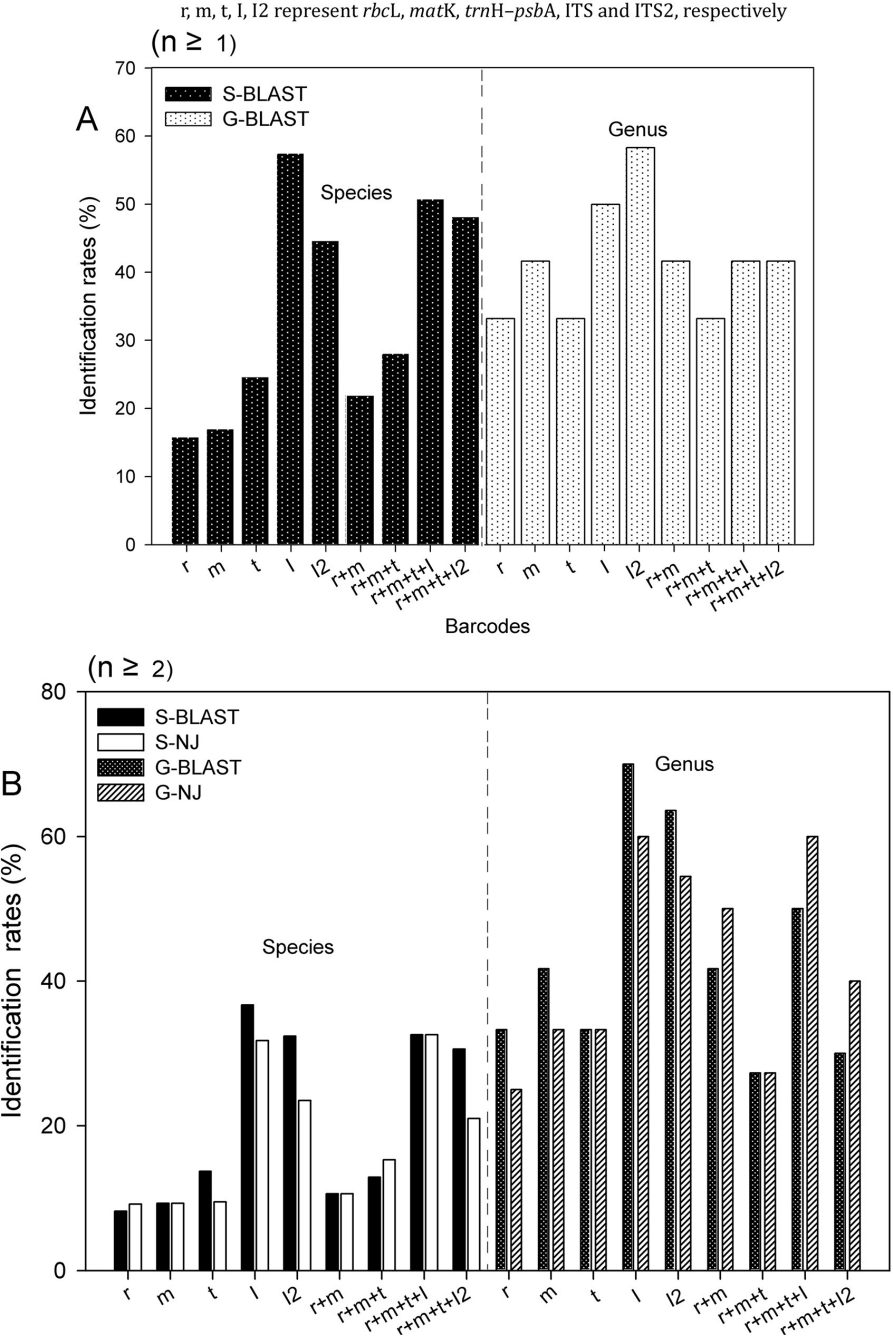
Species resolution at the genus and species levels for single regions and combinations. Results based on BLAST and Neighbor-Joining Tree analyses of the samples (A: n ≥ 1; B: n ≥ 2).

**Fig 4:**
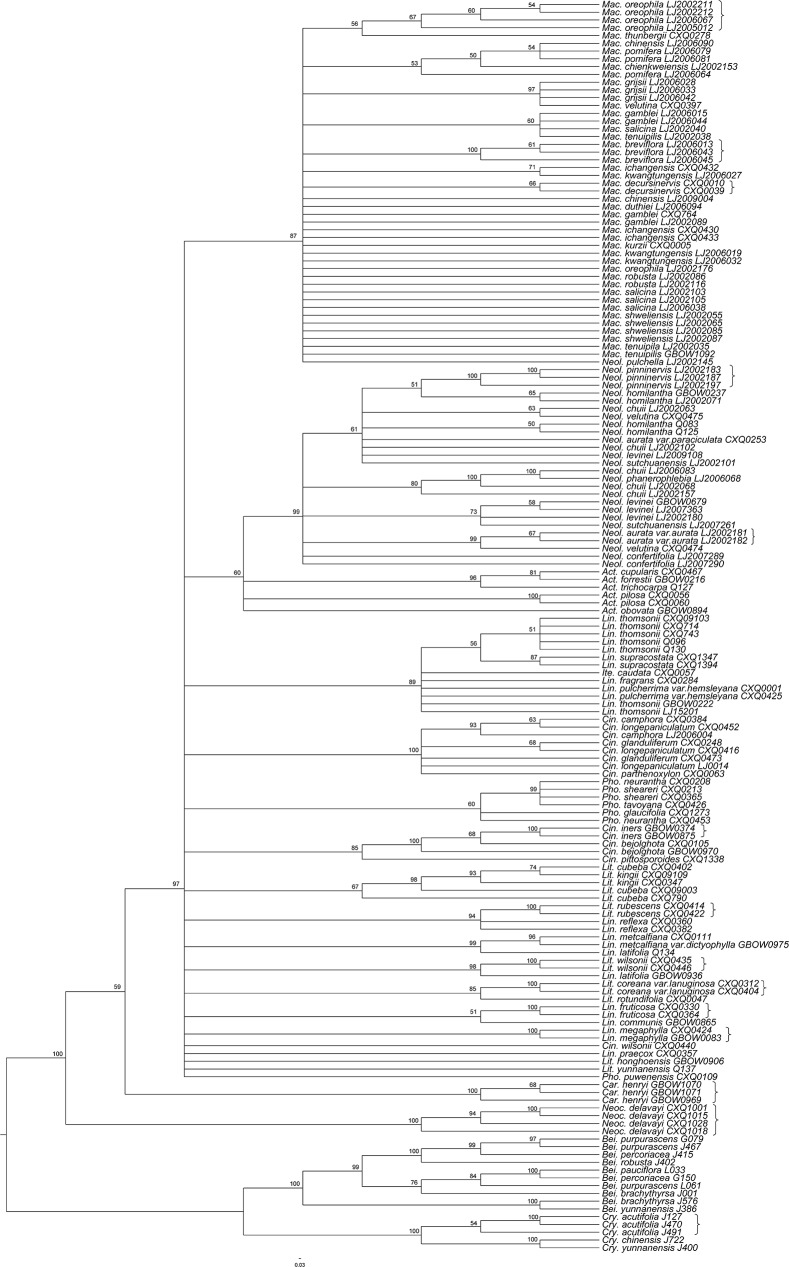
Species resolution success at the genus and species levels for ITS. Result based on Neighbor-Joining Tree analysis (n ≥ 2). The bootstrap values ≥ 50% are shown on the branches. Brackets represent successfully identified species.

After morphological error correction, the resolution rates of species (8.2–57.5%) and genera (25–70%) were calculated, both for individual barcode sequences, as well as for various combinations (Table 1 and Fig 3). For single barcodes, ITS showed the highest discriminatory power of the five markers (Figs 3 and 4), but the discrimination rate was only 57.5% at the species level in BLAST (n ≥ 1) (see Fig 3A). At the genus level, ITS was again the most accurate (70%) in BLAST (n ≥ 2) (see Fig 3B). ITS2 showed lower sequence variation and species discrimination than ITS (see Fig 3A, 44.7% at species level; Fig 3B, 63.6% at genus level), despite its sequence recovery being more or less double that of ITS (Table 1). The discrimination rates of *rbc*L were the lowest (see Fig 3B, 8.2% at species level; 25% at genus level).

Among the marker combinations (*rbc*L+*mat*K, *rbc*L+*mat*K+*trn*H–*psb*A, *rbc*L+*mat*K+*trn*H–*psb*A+ITS and *rbc*L+*mat*K+*trn*H–*psb*A+ITS2), *rbc*L+*mat*K+*trn*H–*psb*A+ITS showed the highest discriminatory power, with discrimination rates of 50.8% at the species level in BLAST (n ≥ 1) (Fig 3A) and 60% at the genus level in NJ Tree (n ≥ 2) (Fig 3B), whereas *rbc*L+*mat*K+*trn*H–*psb*A+ITS2 showed lower discrimination rates (48.2% and 40%) at the same level (Fig 3). In contrast, the combination of *rbc*L+*mat*K showed quite low discrimination rates (10.6%) at species level in both BLAST and NJ Tree (n ≥ 2) (see Fig 3B), but discrimination rate were relatively high (50%) at the genus level in NJ Tree (n ≥ 2) (see Fig 3B). Overall, the tree-based method (NJ Tree) and the similarity-based method (BLAST) provided unsatisfactory discrimination rates.

### Relationships within Lauraceae

**Fig 5:**
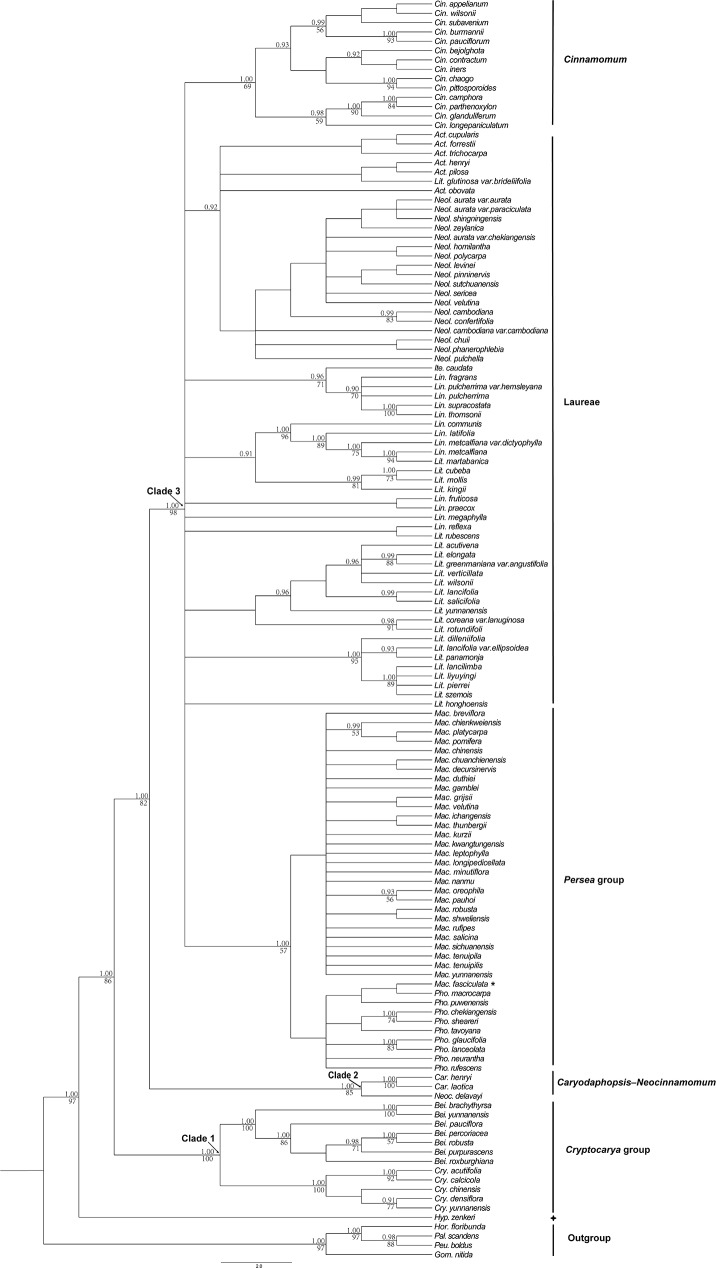
Bayesian consensus tree based on *rbc*L+*mat*K+*trn*H–*psb*A+ITS. Bayesian posterior probabilities (≥ 0.9) / Bootstrap support values (≥ 50%) are shown above the branches. Abbreviations: *Act*. = *Actinodaphne*, *Bei*. = *Beilschmiedia*, *Cin*. = *Cinnamomum*, *Car*. = *Caryodaphnopsis*, *Cry*. = *Cryptocarya*, *Ite*. = *Iteadaphne*, *Lin*. = *Lindera*, *Lit*. = *Litsea*, *Mac*. = *Machilus*, *Neoc*. = *Neocinnamomum*, *Neol*. = *Neolitsea*, *Pho*. = *Phoebe*, *Hyp*. = *Hypodaphnis*, *Hor*. = *Hortonia*, *Pal*. = *Palmeria*, *Peu*. = *Peumus*, *Gom*. = *Gomortega*.

Phylogenetic relationships among 133 species of Lauraceae representing 12 of the 25 genera were analysed after correction. The four DNA markers, *rbc*L, *mat*K, *trn*H–*psb*A and ITS (ITS1+5.8S+ITS2) produced 665, 746, 508 and 845 bp aligned positions respectively, yielding 64, 50, 74, and 149 informative sites and were best fitted to the TVM (Transversional model) +I+G, TIM (Transitional model) +G, K81uf (Two transversion-parameters model 1 unequal frecuencies) +I+G and TrN (Tamura-Nei) +I+G substitution models respectively. As the consensus trees obtained from the BI and MP analyses were almost identical in their topologies, only the Bayesian consensus tree based on *rbc*L+*mat*K+*trn*H–*psb*A+ITS with PP (Posterior Probabilities) and BS values (Bootstrap Support values) is presented here (Fig 5). The Bayesian tree contains three principal Lauraceae clades, with *Hypodaphnis* strongly supported in BI and MP analyses as their sister group. Clade 1 (PP = 1.00, BS = 100%) includes members of the *Cryptocarya* group; *Caryodaphnopsis* and *Neocinnamomum* form Clade 2 (PP = 1.00, BS = 85%); the remainder, representing the *Persea* group, Laureae and *Cinnamomum* formed Clade 3 (PP = 1.00, BS = 98%). In the Clade 3, there is some support for a clade including the representatives of the *Persea* group (PP = 1.00, BS = 57%) and moderate support for a clade including all members of *Cinnamomum* investigated here (PP = 1.00, BS = 69%). All the remaining samples, including members of *Neolitsea*, *Actinodaphne*, *Litsea*, *Lindera* and *Iteadaphne* belong to the Laureae, which do not form a clade in our analysis, but rather a large polytomy of eight clades, plus *Cinnamomum* and the *Persea* group. The outgroup samples, three species of Monimiaceae plus Gomortegaceae also form a monophyletic lineage with strong support (PP = 1.00, BS = 97%).

## Discussion

### Universality of DNA barcodes

Primer universality is an important criterion for a useful DNA barcode [27]. In this regard, the core barcodes (*rbc*L and *mat*K) for Lauraceae plants had the best performance in PCR amplification and sequencing among the five regions (successfully amplifying and sequencing 92.5% individuals), consistent with a previous study [70]. Compared to the above core barcodes, ITS had a relatively low sequencing success rate of 39.1%, because of the lack of universal primers (either published or with potential development by using current information) and poor success by using existing primers [25]. The poor success by using existing primers is probably due largely to the problem of secondary structure formation resulting in poor quality sequence data, multiple copy numbers, etc. [29, 32, 33, 71, 72]. Thus, this region is probably unsuitable as a universal barcode, although it may be useful in particular cases.

### Detecting identification mistakes

Characters such as phyllotaxis, perianth, inflorescence type, size of tepals, or fate of tepals in fruit have been used to delimit the species of Lauraceae [1, 3, 5, 41]. Among these characters, there are some polymorphic characters considered useful at the between-genus level, while they are rarely present together on a specimen when sampled. In *Cryptocarya*, the fruit completely enclosed in the accrescent receptacular is a remarkable character distinguishing it from other genera; however, only some species were flowering when sampled. Hence, *Beilschmiedia purpurascens L061* was wrongly recognized as *Cryptocarya calcicola* (Table 2). Likewise, the persistent and spreading to reflexed tepals in the fruit of *Machilus* are important morphological characters for generic delimitation from the closely related genus *Phoebe*, in which tepals are leathery to woody, conspicuously thickened and clasping the base of the fruit [41]. These characters are also obviously different, but some of these species were also only flowering when sampled, resulting in identification errors, such as *Phoebe tavoyana CXQ0426* (Table 2). There are also some morphological identification errors due to scant information about the species. For example, *Cinnamomum chago* B.S. Sun et H.L. Zhao [73], which had not been included in Flora of China, where if the expert had seen the topotype prior to this study (which has an axillary panicle and short perianth tube), identification errors may not have happened. Furthermore, some genera, such as *Lindera*, *Litsea*, *Neolitsea* and *Actinodaphne*, which form the Laureae, are really not well defined. All the above factors hampered the accurate identification of Lauraceae. Although each sample in the current study is represented by a voucher that was compared to a reference collection, some species often cannot be distinguished in the absence of complete flowering and fruiting material.

DNA barcoding can act as a tool for detecting errors in species identifications [23]. The tree-based and similarity-based approaches using DNA barcoding in combination with morphology are thus very useful to address identification mistakes based only on morphology [22, 74–77]. Examination of the initially misidentified samples showed that misidentifications were most likely to occur when the samples were only flowering or fruiting and their morphological characters and geographical distributions were similar. Once morphology-based errors listed above were taken into account, mistakes in individual identifications were then only detectable through DNA sequencing.

Revision of morphological identifications based mainly on the core barcodes, or the combination of *rbc*L+*mat*K+*trn*H–*psb*A+ITS, supplemented by BLAST analyses, determined that 10.8% individuals had been misidentified *a priori* based on morphology (Table 2). This error rate is higher than those reported for some other studies (5.6–10.5%, Archaux *et al*. [78]; 7.4%, Scott & Hallam [79]; 6.8–7.6%, Dexter *et al*. [17]; 9.9%, Huang *et al*. [22]), suggesting that the Lauraceae require careful interpretation of the characters used for specific and generic definition. In particular, accurate recognition of Lauraceae would be very useful because it is the most diverse family in China and is known to be taxonomically problematic.

### Evaluation of DNA barcodes for Lauraceae

Our study gives a reliable assessment of barcoding efficacy in the family Lauraceae based on a large sample size, comparable to the results of studies for other diverse angiosperm groups (*e*.*g*., Percy *et al*.: 77 species of *Salix* [15]; Edwards *et al*.: 82 species of *Aspalathus* [80]; Yu *et al*.: 88 species of *Pedicularis* [81]). An ideal DNA barcode must combine conserved regions for universal primer design, which show high rates of PCR amplification and sequencing [28] and should also provide a high rate of success for species discrimination and identification [25, 30, 82].

In the present study, the five barcodes performed differently for all samples (Table 1 and Fig 3) and out of all regions tested, ITS performed best, showing the greatest level of species discrimination. However, other studies have described inherent difficulties with this marker [29, 32, 33, 71, 72] and some researchers have advocated using ITS2 alone as a replacement for ITS because it is easier to amplify and sequence this subset of the marker [32, 33]. In contrast, ITS2 showed lower sequence variation and species identification ability than ITS in our study, even though its sequence recovery rate is about two times that of ITS, but we did not observe the other difficulties usually associated with ITS as a barcode marker, so the marker appears to have potential for Lauraceae as long as the low sequencing success rate can be addressed.

ITS was proposed as a DNA barcode for seed plants because of its high species identification ability [25, 33] and in this study ITS provided the highest species resolution, agreeing with the results of recent studies in other plant groups (*e*.*g*., Poaceae: Cai *et al*. [83]; Schisandraceae: Zhang *et al*. [84]; Orchidaceae: Li *et al*. [85]). The other four barcoding regions investigated here (*rbc*L, *mat*K, *trn*H–*psb*A and ITS2 alone) have all been proposed as core or supplementary regions for plant barcoding [25, 28, 29,32, 82, 86], but in our study they exhibited low species-level resolution and only *Cryptocarya* and *Beilschmiedia* were distinguished clearly from the other genera. This suggests that ITS is the best candidate for Lauraceae when using a single barcode.

Combining DNA barcodes is generally considered to improve species identification [28, 33, 87, 88] and in this study, the discrimination rates of the combinations varied from 10.6% to 32.6% with *rbc*L+*mat*K < *rbc*L+*mat*K+*trn*H–*psb*A < *rbc*L+*mat*K+*trn*H–*psb*A+ITS2 < *rbc*L+*mat*K+*trn*H–*psb*A+ITS at the species level (Fig 3). However, we can see that the discrimination rates of *rbc*L+*mat*K are higher than those of *rbc*L+*mat*K+*trn*H–*psb*A and *rbc*L+*mat*K+*trn*H–*psb*A+ITS2 at the genus level. The utility of a marker is not only affected by its discriminatory power, but also by its rate of sequence recovery (Figs 2–5).

Species delimitation in Lauraceae is often complicated by a lack of unique qualitative morphological characters that can be used to define them. DNA barcode data can therefore provide useful additional information for evaluation of observed morphological diversity [89]. Efficient species identification is also important for customs and other authorities to prevent the illegal export and commercial use of protected or rare species [90]. Thus, it is suggested here that using ITS as single barcode, or a combination of barcode markers that included ITS, would be the most suitable approach for barcoding in Lauraceae.

### Relationships among major clades

The BI and MP analyses provided relatively good phylogenetic resolution for Lauraceae at both generic and intrageneric levels (Fig 5), especially in basal lineages, with the *Cryptocarya* group, the *Caryodaphnopsis*–*Neocinnamomum* group and the *Persea* group plus Laureae and *Cinnamomum* corresponding to our Clades 1, 2 and 3 respectively. Within the *Cryptocarya* group, which is basal within Lauraceae [7, 47], *Cryptocarya* is sister to the non-cupulate clade of *Beilschmiedia*. *Cryptocarya* has a deeply urceolate floral hypanthium that develops into a deep cupule enclosing the drupe at maturity, except for a small terminal orifice [7, 46], but *Beilschmiedia* lacks these characters; a synapomorphy that separates *Beilschmiedia* and related genera (*Endiandra* and *Syndiclis*) from the rest of the *Cryptocarya* group.

*Caryodaphnopsis* and *Neocinnamomum* are associated in the present study and have been found previously to have a relatively close relationship [47, 91, 92]. They share triplinerved venation and four-locular anthers with the loculi arranged in a shallow arc [7], sometimes two-locular in *Caryodaphnopsis*, or in a horizontal row, such as in *Neocinnamomum delavayi* (Lecomte) H. Liu.

The remaining clade (the *Persea* group, Laureae and *Cinnamomum*) with *Machilus* and *Phoebe* as subsets of the *Persea* group received moderate support, agreeing with the studies of Chanderbali *et al*. [7], Li *et al*. [41] and Rohwer *et al*. [44]. However, as with these earlier studies, there was poor resolution for species relationships within *Machilus* and its presently accepted sections and subsections (*e*.*g*. Li *et al*. [93]) are still questionable. Nevertheless, the present study does suggest that *M*. *fasciculata* H. W. Li belongs in *Phoebe*. *Cinnamomum* was divided into two clades corresponding to sect. *Camphora* Meissn. and sect. *Cinnamomum* [63], reflecting morphological traits such as leaf arrangement, leaf venation pattern, presence or absence of perulate buds or domatia.

The remaining sampled Laureae were poorly resolved, even though a close relationship between *Actinodaphne*, *Lindera*, *Litsea* and *Neolitsea* has been recognized in almost all Lauraceae classifications [7]. All of these genera are dioecious and most have umbellate inflorescences subtended by involucral bracts [7], but further character evolution study is needed to determine if these features actually represent synplesiomorphies. This suggests that although multilocus molecular markers still do not give well-resolved phylogenies for all Lauraceae, DNA barcoding is nevertheless useful for resolving phylogenetic relationships at the generic or species level within some groups in the family.

## Conclusions

The barcodes used here produced positive results for correcting species identification errors and reconstructing phylogenetic relationships of Lauraceae, even though identification rates were not high. Furthermore, because DNA barcoding plays an important role in the conservation of rare species and for forest crime prosecutions, we advocate the use of DNA barcodes, in combination with other techniques, in order to develop adequate management strategies for the long term conservation of Lauraceae. In particular, barcodes such as ITS show promise for large-scale biodiversity assessment and inventory, particularly for tropical tree species, where the use of a single barcode could significantly reduce the time and costs involved with species identification. However, our study also indicates the critical need for additional data from both more taxa and more sequence regions to help resolve issues in Lauraceae taxonomy and conservation, as there is clearly no simple one-size-fits-all barcoding solution for the family.

## Supporting information

S1 Table Taxa, voucher specimens, the locations and geographic coordinates in this study. (XLSX)

S2 Table Summary of the sequence recovery of five DNA barcode regions and GenBank accession numbers. (DOCX)

S3 Table The systems and reaction processes of the PCR amplification protocols used for barcoding. (DOCX)

S1 Fig Neighbor-Joining (NJ) tree generated using *rbcL* sequences. Erroneous identifications which recognized based on *rbc*L marker at the genus and species levels are marked by stars.
(TIFF)

S2 Fig Neighbor-Joining (NJ) tree generated using *mat*K sequences. Erroneous identifications which recognized based on *mat*K marker at the genus and species levels are marked by stars.
(TIFF)
