# Supplementary material for: DNA barcoding evaluation and implications for phylogenetic relationships in Lauraceae from China
Source: PLoS One. 2017 Apr 17;12(4):e0175788. doi: 10.1371/journal.pone.0175788 (PMC5393608; doi:10.1371/journal.pone.0175788)
Supplement: S2 Table — (DOCX) [file pone.0175788.s002.docx]

**S2 Table Summary of the sequence recovery of five DNA barcode regions and GenBank accession numbers**

| **Taxon** | | **Herbarium/ Voucher number** | **GenBank Accession Numbers** | | | | |
| --- | --- | --- | --- | --- | --- | --- | --- |
|  |  |  | **rbcL** | **matK** | **trnH-psbA** | **ITS** | **ITS2** |
| Lauraceae | *Actinodaphne cupularis* | HITBC CXQ0443 | KX546783 | KX545690 | KX546071 |  |  |
| Lauraceae | *Actinodaphne cupularis* | HITBC CXQ0454 | KX546784 | KX545691 | KX546072 |  | KX546555 |
| Lauraceae | *Actinodaphne cupularis* | HITBC CXQ0467 | KX546785 | KX545692 | KX546073 | KX546394 | KX546556 |
| Lauraceae | *Actinodaphne forrestii* | HITBC GBOW0091 | KX546786 | KX545693 | KX546074 |  | KX546557 |
| Lauraceae | *Actinodaphne forrestii* | HITBC GBOW0216 | KX546787 | KX545694 | KX546075 | KX546395 | KX546558 |
| Lauraceae | *Actinodaphne forrestii* | HITBC GBOW0250 | KX546788 | KX545695 |  |  | KX546559 |
| Lauraceae | *Actinodaphne forrestii* | HITBC Q141 | KX546789 | KX545696 | KX546076 |  |  |
| Lauraceae | *Actinodaphne henryi* | HITBC J066 | KX546790 |  |  |  |  |
| Lauraceae | *Actinodaphne obovata* | HITBC GBOW0400 | KX546791 | KX545697 | KX546077 |  | KX546560 |
| Lauraceae | *Actinodaphne obovata* | HITBC GBOW0894 | KX546792 | KX545698 | KX546078 | KX546396 | KX546561 |
| Lauraceae | *Actinodaphne pilosa* | HITBC CXQ0056 | KX546793 | KX545699 | KX546079 | KX546397 | KX546562 |
| Lauraceae | *Actinodaphne pilosa* | HITBC CXQ0060 | KX546794 | KX545700 | KX546080 | KX546398 | KX546563 |
| Lauraceae | *Actinodaphne trichocarpa* | HITBC Q127 | KX546795 | KX545701 | KX546081 | KX546399 | KX546564 |
| Lauraceae | *Beilschmiedia brachythyrsa* | HITBC J001 |  |  |  | KX546400 | KX546565 |
| Lauraceae | *Beilschmiedia brachythyrsa* | HITBC J316 | KX546796 | KX545702 |  |  |  |
| Lauraceae | *Beilschmiedia brachythyrsa* | HITBC J576 | KX546797 | KX545703 |  | KX546401 | KX546566 |
| Lauraceae | *Beilschmiedia brachythyrsa* | HITBC L026 |  |  |  |  | KX546567 |
| Lauraceae | *Beilschmiedia pauciflora* | HITBC L033 |  |  |  | KX546402 | KX546568 |
| Lauraceae | *Beilschmiedia percoriacea* | HITBC G148 | KX546798 | KX545704 |  |  |  |
| Lauraceae | *Beilschmiedia percoriacea* | HITBC G150 | KX546799 | KX545705 | KX546082 | KX546403 | KX546569 |
| Lauraceae | *Beilschmiedia percoriacea* | HITBC J291 | KX546800 | KX545706 |  |  | KX546570 |
| Lauraceae | *Beilschmiedia percoriacea* | HITBC J415 | KX546801 | KX545707 | KX546083 | KX546404 | KX546571 |
| Lauraceae | *Beilschmiedia purpurascens* | HITBC G079 | KX546802 | KX545708 | KX546084 | KX546405 | KX546572 |
| Lauraceae | *Beilschmiedia purpurascens* | HITBC G165 | KX546803 | KX545709 |  |  | KX546573 |
| Lauraceae | *Beilschmiedia purpurascens* | HITBC G174 | KX546804 | KX545710 | KX546085 |  |  |
| Lauraceae | *Beilschmiedia purpurascens* | HITBC J032 | KX546805 |  | KX546086 |  | KX546574 |
| Lauraceae | *Beilschmiedia purpurascens* | HITBC J467 | KX546806 | KX545711 |  | KX546406 | KX546578 |
| Lauraceae | *Beilschmiedia purpurascens* | HITBC J528 | KX546807 | KX545712 |  |  |  |
| Lauraceae | *Beilschmiedia purpurascens* | HITBC L061 |  |  |  | KX546407 | KX546576 |
| Lauraceae | *Beilschmiedia robusta* | HITBC J402 | KX546808 | KX545713 | KX546087 | KX546408 |  |
| Lauraceae | *Beilschmiedia robusta* | HITBC J711 | KX546809 | KX545714 | KX546088 |  |  |
| Lauraceae | *Beilschmiedia robusta* | HITBC CXQ0279 |  |  |  |  | KX546575 |
| Lauraceae | *Beilschmiedia roxburghiana* | HITBC G065 | KX546810 | KX545715 | KX546089 |  |  |
| Lauraceae | *Beilschmiedia roxburghiana* | HITBC G188 | KX546811 | KX545716 | KX546090 |  |  |
| Lauraceae | *Beilschmiedia roxburghiana* | HITBC J677 | KX546812 |  |  |  |  |
| Lauraceae | *Beilschmiedia yunnanensis* | HITBC J088 | KX546813 |  |  |  |  |
| Lauraceae | *Beilschmiedia yunnanensis* | HITBC J193 | KX546814 | KX545717 |  |  |  |
| Lauraceae | *Beilschmiedia yunnanensis* | HITBC J386 | KX546815 |  |  | KX546409 | KX546577 |
| Lauraceae | *Beilschmiedia yunnanensis* | HITBC J485 |  | KX545718 |  |  |  |
| Lauraceae | *Caryodaphnopsis henryi* | HITBC GBOW0969 | KX546816 | KX545719 |  | KX546410 | KX546579 |
| Lauraceae | *Caryodaphnopsis henryi* | HITBC GBOW1070 | KX546817 | KX545720 | KX546091 | KX546411 | KX546580 |
| Lauraceae | *Caryodaphnopsis henryi* | HITBC GBOW1071 | KX546818 | KX545721 | KX546092 | KX546412 | KX546581 |
| Lauraceae | *Caryodaphnopsis laotica* | HITBC GBOW0745 | KX546819 | KX545722 |  |  |  |
| Lauraceae | *Cinnamomum appelianum* | HITBC CXQ0203 | KX546820 | KX545723 | KX546093 |  | KX546582 |
| Lauraceae | *Cinnamomum appelianum* | HITBC CXQ0204 | KX546821 | KX545724 | KX546094 |  | KX546583 |
| Lauraceae | *Cinnamomum appelianum* | HITBC CXQ09105 | KX546822 | KX545725 | KX546095 |  | KX546584 |
| Lauraceae | *Cinnamomum bejolghota* | HITBC CXQ0105 | KX546823 | KX545726 | KX546096 | KX546413 | KX546585 |
| Lauraceae | *Cinnamomum bejolghota* | HITBC GBOW0970 | KX546824 | KX545727 |  | KX546414 | KX546586 |
| Lauraceae | *Cinnamomum bejolghota* | HITBC J559 | KX546825 | KX545728 |  |  |  |
| Lauraceae | *Cinnamomum burmannii* | HITBC CXQ0020 | KX546826 | KX545729 | KX546097 |  |  |
| Lauraceae | *Cinnamomum burmannii* | HITBC CXQ0368 | KX546827 | KX545730 | KX546098 |  |  |
| Lauraceae | *Cinnamomum burmannii* | HITBC CXQ09025 | KX546828 | KX545731 | KX546099 |  |  |
| Lauraceae | *Cinnamomum camphora* | HITBC CXQ0224 | KX546829 | KX545732 | KX546100 |  |  |
| Lauraceae | *Cinnamomum camphora* | HITBC CXQ0384 | KX546830 | KX545733 | KX546101 | KX546415 | KX546587 |
| Lauraceae | *Cinnamomum camphora* | HITBC CXQ0405 | KX546831 | KX545734 | KX546102 |  |  |
| Lauraceae | *Cinnamomum camphora* | HITBC LJ2006004 | KX546832 | KX545735 | KX546103 | KX546416 | KX546588 |
| Lauraceae | *Cinnamomum chago* | HITBC CXQ690 | KX546833 |  | KX546104 |  |  |
| Lauraceae | *Cinnamomum chago* | HITBC CXQ762 |  | KX545736 |  |  |  |
| Lauraceae | *Cinnamomum chago* | HITBC LJ2002064 | KX546834 | KX545737 | KX546105 |  |  |
| Lauraceae | *Cinnamomum chago* | HITBC LJ2002072 | KX546835 |  | KX546106 |  |  |
| Lauraceae | *Cinnamomum chago* | HITBC Q090 | KX546836 | KX545738 | KX546107 |  |  |
| Lauraceae | *Cinnamomum chago* | HITBC Q129 |  | KX545739 | KX546108 |  |  |
| Lauraceae | *Cinnamomum contractum* | HITBC G023 |  |  | KX546109 |  |  |
| Lauraceae | *Cinnamomum contractum* | HITBC J336 | KX546837 | KX545740 |  |  |  |
| Lauraceae | *Cinnamomum contractum* | HITBC J443 | KX546838 |  |  |  |  |
| Lauraceae | *Cinnamomum glanduliferum* | HITBC CXQ0248 | KX546839 | KX545741 | KX546110 | KX546417 | KX546589 |
| Lauraceae | *Cinnamomum glanduliferum* | HITBC CXQ0473 | KX546840 |  | KX546111 | KX546418 | KX546590 |
| Lauraceae | *Cinnamomum iners* | HITBC CXQ0077 | KX546841 | KX545742 | KX546112 |  |  |
| Lauraceae | *Cinnamomum iners* | HITBC GBOW0374 | KX546842 | KX545743 | KX546113 | KX546419 | KX546591 |
| Lauraceae | *Cinnamomum iners* | HITBC GBOW0875 | KX546843 | KX545744 | KX546114 | KX546420 | KX546592 |
| Lauraceae | *Cinnamomum longepaniculatum* | HITBC CXQ0416 | KX546844 | KX545745 | KX546115 | KX546421 | KX546593 |
| Lauraceae | *Cinnamomum longepaniculatum* | HITBC CXQ0437 | KX546845 | KX545746 | KX546116 |  |  |
| Lauraceae | *Cinnamomum longepaniculatum* | HITBC CXQ0452 | KX546846 | KX545747 | KX546117 | KX546422 | KX546594 |
| Lauraceae | *Cinnamomum longepaniculatum* | HITBC LJ0014 | KX546847 | KX545748 | KX546118 | KX546423 | KX546595 |
| Lauraceae | *Cinnamomum parthenoxylon* | HITBC CXQ0063 | KX546848 | KX545749 | KX546119 | KX546424 | KX546596 |
| Lauraceae | *Cinnamomum parthenoxylon* | HITBC CXQ0255 | KX546849 | KX545750 | KX546120 | KX546425 |  |
| Lauraceae | *Cinnamomum parthenoxylon* | HITBC CXQ09001 | KX546850 | KX545751 | KX546121 |  | KX546597 |
| Lauraceae | *Cinnamomum pauciflorum* | HITBC CXQ0040 | KX546851 | KX545752 | KX546122 | KX546426 |  |
| Lauraceae | *Cinnamomum pauciflorum* | HITBC CXQ0202 | KX546852 | KX545753 | KX546123 |  | KX546598 |
| Lauraceae | *Cinnamomum pauciflorum* | HITBC CXQ0287 | KX546853 | KX545754 | KX546124 |  | KX546599 |
| Lauraceae | *Cinnamomum pittosporoides* | HITBC CXQ1338 | KX546854 | KX545755 | KX546125 |  | KX546600 |
| Lauraceae | *Cinnamomum subavenium* | HITBC CXQ0017 | KX546855 | KX545756 | KX546126 |  |  |
| Lauraceae | *Cinnamomum subavenium* | HITBC CXQ0257 | KX546856 | KX545757 | KX546127 |  |  |
| Lauraceae | *Cinnamomum subavenium* | HITBC CXQ0321 | KX546857 | KX545758 | KX546128 |  |  |
| Lauraceae | *Cinnamomum wilsonii* | HITBC CXQ0206 | KX546858 | KX545759 | KX546129 |  |  |
| Lauraceae | *Cinnamomum wilsonii* | HITBC CXQ0440 | KX546859 | KX545760 | KX546130 |  | KX546601 |
| Lauraceae | *Cryptocarya acutifolia* | HITBC J116 | KX546860 |  |  |  |  |
| Lauraceae | *Cryptocarya acutifolia* | HITBC J127 |  | KX545761 |  | KX546427 | KX546602 |
| Lauraceae | *Cryptocarya acutifolia* | HITBC J135 | KX546861 |  |  |  |  |
| Lauraceae | *Cryptocarya acutifolia* | HITBC J470 | KX546862 | KX545762 | KX546131 | KX546428 | KX546603 |
| Lauraceae | *Cryptocarya acutifolia* | HITBC J491 | KX546863 | KX545763 | KX546132 | KX546429 | KX546604 |
| Lauraceae | *Cryptocarya acutifolia* | HITBC J537 | KX546864 | KX545764 |  |  |  |
| Lauraceae | *Cryptocarya acutifolia* | HITBC J542 | KX546865 | KX545765 | KX546133 |  |  |
| Lauraceae | *Cryptocarya calcicola* | HITBC CXQ0069 | KX546866 | KX545766 |  |  |  |
| Lauraceae | *Cryptocarya acutifolia* | HITBC J607 | KX546867 | KX545767 |  |  |  |
| Lauraceae | *Cryptocarya calcicola* | HITBC L050 | KX546868 |  |  |  |  |
| Lauraceae | *Cryptocarya chinensis* | HITBC J722 | KX546869 | KX545768 | KX546134 | KX546430 | KX546605 |
| Lauraceae | *Cryptocarya densiflora* | HITBC GBOW0548 | KX546870 | KX545769 | KX546135 |  |  |
| Lauraceae | *Cryptocarya densiflora* | HITBC J600 | KX546871 | KX545770 |  |  |  |
| Lauraceae | *Cryptocarya yunnanensis* | HITBC J198 | KX546872 |  |  |  |  |
| Lauraceae | *Cryptocarya yunnanensis* | HITBC J400 |  | KX545771 |  | KX546431 | KX546606 |
| Lauraceae | *Iteadaphne caudata* | HITBC CXQ0057 | KX546873 | KX545772 | KX546136 | KX546432 | KX546607 |
| Lauraceae | *Iteadaphne caudata* | HITBC CXQ09004 | KX546874 | KX545773 | KX546137 |  | KX546608 |
| Lauraceae | *Iteadaphne caudata* | HITBC CXQ09006 |  | KX545774 |  |  |  |
| Lauraceae | *Iteadaphne caudata* | HITBC CXQ1029 | KX546875 | KX545775 |  |  |  |
| Lauraceae | *Iteadaphne caudata* | HITBC GBOW0825 | KX546876 | KX545776 | KX546138 |  |  |
| Lauraceae | *Lindera communis* | HITBC CXQ0215 | KX546877 |  | KX546139 |  | KX546609 |
| Lauraceae | *Lindera communis* | HITBC CXQ09005 | KX546878 | KX545777 | KX546140 |  | KX546610 |
| Lauraceae | *Lindera communis* | HITBC GBOW0865 | KX546879 | KX545778 | KX546141 | KX546433 | KX546611 |
| Lauraceae | *Lindera fragrans* | HITBC CXQ0284 | KX546880 | KX545779 | KX546142 | KX546434 | KX546612 |
| Lauraceae | *Lindera fruticosa* | HITBC CXQ0330 | KX546881 | KX545780 | KX546143 | KX546435 | KX546613 |
| Lauraceae | *Lindera fruticosa* | HITBC CXQ0364 | KX546882 | KX545781 | KX546144 | KX546436 | KX546614 |
| Lauraceae | *Lindera latifolia* | HITBC CXQ1108 | KX546883 | KX545782 | KX546145 |  |  |
| Lauraceae | *Lindera latifolia* | HITBC CXQ1120 | KX546884 | KX545783 |  |  |  |
| Lauraceae | *Lindera latifolia* | HITBC Q134 | KX546885 | KX545784 | KX546146 | KX546437 | KX546616 |
| Lauraceae | *Lindera megaphylla* | HITBC CXQ0424 | KX546886 | KX545786 | KX546147 | KX546438 | KX546617 |
| Lauraceae | *Lindera megaphylla* | HITBC CXQ0466 | KX546887 | KX545787 | KX546148 |  | KX546618 |
| Lauraceae | *Lindera megaphylla* | HITBC GBOW0083 | KX546888 | KX545788 | KX546149 | KX546439 | KX546619 |
| Lauraceae | *Lindera metcalfiana* | HITBC CXQ0111 | KX546889 | KX545789 | KX546150 | KX546440 | KX546620 |
| Lauraceae | *Lindera metcalfiana* | HITBC CXQ09063 | KX546890 | KX545790 | KX546151 |  | KX546621 |
| Lauraceae | *Lindera metcalfiana* | HITBC CXQ09068 | KX546891 | KX545791 | KX546152 |  | KX546622 |
| Lauraceae | *Lindera metcalfiana* | HITBC G145 |  | KX545792 | KX546153 |  |  |
| Lauraceae | *Lindera metcalfiana* | HITBC J330 |  | KX545793 |  |  |  |
| Lauraceae | *Lindera metcalfiana* | HITBC J572 | KX546892 | KX545794 |  |  |  |
| Lauraceae | *Lindera metcalfiana var. dictyophylla* | HITBC J721 | KX546893 | KX545795 | KX546154 |  |  |
| Lauraceae | *Lindera metcalfiana var. dictyophylla* | HITBC GBOW0336 | KX546894 | KX545796 | KX546155 |  | KX546623 |
| Lauraceae | *Lindera metcalfiana var. dictyophylla* | HITBC GBOW0547 | KX546895 | KX545797 | KX546156 |  | KX546624 |
| Lauraceae | *Lindera metcalfiana var. dictyophylla* | HITBC GBOW0975 | KX546896 | KX545798 | KX546157 | KX546441 | KX546625 |
| Lauraceae | *Lindera praecox* | HITBC CXQ0320 | KX546897 | KX545799 | KX546158 |  |  |
| Lauraceae | *Lindera praecox* | HITBC CXQ0357 | KX546898 | KX545800 | KX546159 | KX546442 | KX546626 |
| Lauraceae | *Lindera praecox* | HITBC CXQ0395 | KX546899 | KX545801 | KX546160 |  | KX546627 |
| Lauraceae | *Lindera pulcherrima var.hemsleyana* | HITBC CXQ0001 | KX546900 | KX545802 | KX546161 | KX546443 | KX546628 |
| Lauraceae | *Lindera pulcherrima var.hemsleyana* | HITBC CXQ0425 | KX546901 | KX545803 | KX546162 | KX546444 | KX546629 |
| Lauraceae | *Lindera pulcherrima var.hemsleyana* | HITBC CXQ0468 | KX546902 | KX545804 | KX546163 |  | KX546630 |
| Lauraceae | *Lindera reflexa* | HITBC CXQ0222 | KX546903 | KX545805 | KX546164 |  |  |
| Lauraceae | *Lindera reflexa* | HITBC CXQ0360 | KX546904 | KX545806 | KX546165 | KX546445 | KX546631 |
| Lauraceae | *Lindera reflexa* | HITBC CXQ0382 | KX546905 | KX545807 | KX546166 | KX546446 | KX546632 |
| Lauraceae | *Lindera supracostata* | HITBC CXQ1347 | KX546906 | KX545808 | KX546167 | KX546447 | KX546633 |
| Lauraceae | *Lindera supracostata* | HITBC CXQ1394 | KX546907 | KX545809 | KX546168 | KX546448 | KX546634 |
| Lauraceae | *Lindera supracostata* | HITBC CXQ1398 | KX546908 | KX545810 | KX546169 | KX546449 |  |
| Lauraceae | *Lindera thomsonii* | HITBC CXQ09103 | KX546909 | KX545811 |  |  | KX546635 |
| Lauraceae | *Lindera thomsonii* | HITBC CXQ714 | KX546910 | KX545812 | KX546170 | KX546450 | KX546636 |
| Lauraceae | *Lindera thomsonii* | HITBC CXQ743 | KX546911 | KX545813 | KX546171 | KX546451 | KX546637 |
| Lauraceae | *Lindera thomsonii* | HITBC GBOW0222 | KX546912 | KX545814 | KX546172 | KX546452 | KX546638 |
| Lauraceae | *Lindera thomsonii* | HITBC LJ15201 | KX546913 | KX545815 | KX546173 | KX546453 | KX546639 |
| Lauraceae | *Lindera thomsonii* | HITBC Q096 | KX546914 | KX545816 | KX546174 | KX546454 | KX546640 |
| Lauraceae | *Lindera thomsonii* | HITBC Q130 | KX546915 | KX545817 | KX546175 | KX546455 | KX546641 |
| Lauraceae | *Litsea acutivena* | HITBC J092 | KX546916 | KX545818 | KX546176 |  |  |
| Lauraceae | *Litsea acutivena* | HITBC J083 | KX546917 |  |  |  |  |
| Lauraceae | *Litsea coreana var. lanuginosa* | HITBC CXQ0312 | KX546918 | KX545819 | KX546177 | KX546456 | KX546642 |
| Lauraceae | *Litsea coreana var. lanuginosa* | HITBC CXQ0356 | KX546919 | KX545820 | KX546178 |  |  |
| Lauraceae | *Litsea coreana var. lanuginosa* | HITBC CXQ0404 | KX546920 | KX545821 | KX546179 | KX546457 | KX546643 |
| Lauraceae | *Litsea cubeba* | HITBC CXQ0247 | KX546921 | KX545822 | KX546180 |  | KX546644 |
| Lauraceae | *Litsea cubeba* | HITBC CXQ0402 | KX546922 | KX545823 | KX546181 | KX546458 | KX546645 |
| Lauraceae | *Litsea cubeba* | HITBC CXQ09003 | KX546923 | KX545824 | KX546182 | KX546459 | KX546646 |
| Lauraceae | *Litsea cubeba* | HITBC CXQ790 | KX546924 | KX545825 | KX546183 | KX546460 | KX546647 |
| Lauraceae | *Litsea dilleniifolia* | HITBC G107 | KX546925 | KX545826 | KX546184 |  |  |
| Lauraceae | *Litsea elongata* | HITBC CXQ0002 | KX546926 | KX545827 | KX546185 |  | KX546648 |
| Lauraceae | *Litsea elongata* | HITBC CXQ0266 | KX546927 | KX545828 | KX546186 |  | KX546649 |
| Lauraceae | *Litsea elongata* | HITBC CXQ09059 | KX546928 | KX545829 | KX546187 |  | KX546650 |
| Lauraceae | *Litsea elongata* | HITBC G196 | KX546929 | KX545830 |  |  |  |
| Lauraceae | *Litsea elongata* | HITBC J515 | KX546930 | KX545831 |  |  |  |
| Lauraceae | *Litsea elongata* | HITBC J668 | KX546931 | KX545832 |  |  |  |
| Lauraceae | *Litsea elongata* | HITBC Q071 | KX546932 | KX545833 |  |  |  |
| Lauraceae | *Litsea elongata* | HITBC Q126 | KX546933 | KX545834 | KX546188 |  |  |
| Lauraceae | *Litsea elongata* | HITBC Q147 | KX546934 | KX545835 | KX546189 |  |  |
| Lauraceae | *Litsea elongata var. subverticillata* | HITBC J133 | KX546935 |  |  |  |  |
| Lauraceae | *Litsea glutinosa var.brideliifolia* | HITBC CXQ0051 | KX546936 | KX545836 | KX546190 |  |  |
| Lauraceae | *Litsea glutinosa var.brideliifolia* | HITBC CXQ0053 | KX546937 | KX545837 | KX546191 |  | KX546651 |
| Lauraceae | *Litsea greenmaniana var. angustifolia* | HITBC CXQ0080 | KX546938 | KX545838 | KX546192 |  | KX546652 |
| Lauraceae | *Litsea greenmaniana var. angustifolia* | HITBC Q133 | KX546939 | KX545839 | KX546193 |  |  |
| Lauraceae | *Litsea honghoensis* | HITBC CXQ09007 | KX546940 | KX545840 | KX546194 |  |  |
| Lauraceae | *Litsea honghoensis* | HITBC CXQ09051 | KX546941 | KX545841 | KX546195 |  |  |
| Lauraceae | *Litsea honghoensis* | HITBC GBOW0906 | KX546942 | KX545842 | KX546196 | KX546461 | KX546653 |
| Lauraceae | *Litsea honghoensis* | HITBC Q085 | KX546943 | KX545843 | KX546197 |  |  |
| Lauraceae | *Litsea honghoensis* | HITBC Q131 | KX546944 | KX545844 | KX546198 |  |  |
| Lauraceae | *Litsea kingii* | HITBC CXQ0075 | KX546945 | KX545845 | KX546199 |  |  |
| Lauraceae | *Litsea kingii* | HITBC CXQ0347 | KX546946 | KX545846 | KX546200 | KX546462 | KX546654 |
| Lauraceae | *Litsea kingii* | HITBC CXQ09109 | KX546947 | KX545847 | KX546201 | KX546463 | KX546655 |
| Lauraceae | *Litsea lancifolia* | HITBC J409 | KX546948 | KX545848 | KX546202 |  |  |
| Lauraceae | *Litsea lancifolia var. ellipsoidea* | HITBC GBOW0779 | KX546949 | KX545849 | KX546203 |  |  |
| Lauraceae | *Litsea lancifolia var. ellipsoidea* | HITBC GBOW0985 | KX546950 | KX545850 | KX546204 |  |  |
| Lauraceae | *Litsea lancifolia var. ellipsoidea* | HITBC GBOW1343 | KX546951 | KX545851 | KX546205 |  | KX546656 |
| Lauraceae | *Litsea lancilimba* | HITBC J407 | KX546952 | KX545852 |  |  |  |
| Lauraceae | *Litsea liyuyingi* | HITBC J199 | KX546953 | KX545853 |  |  |  |
| Lauraceae | *Litsea liyuyingi* | HITBC J366 | KX546954 | KX545854 |  |  |  |
| Lauraceae | *Litsea liyuyingi* | HITBC J371 | KX546955 | KX545855 |  |  |  |
| Lauraceae | *Litsea martabanica* | HITBC CXQ1113 | KX546956 | KX545856 | KX546206 |  |  |
| Lauraceae | *Litsea martabanica* | HITBC CXQ1271 | KX546957 | KX545857 | KX546207 |  |  |
| Lauraceae | *Litsea martabanica* | HITBC G090 |  | KX545858 | KX546208 |  |  |
| Lauraceae | *Litsea mollis* | HITBC CXQ0227 | KX546958 | KX545859 | KX546209 |  |  |
| Lauraceae | *Litsea mollis* | HITBC CXQ0234 | KX546959 | KX545860 | KX546210 |  | KX546657 |
| Lauraceae | *Litsea mollis* | HITBC GBOW1060 | KX546960 | KX545861 | KX546211 |  | KX546658 |
| Lauraceae | *Litsea mollis* | HITBC J667 | KX546961 | KX545862 |  |  |  |
| Lauraceae | *Litsea panamonja* | HITBC J003 | KX546962 |  |  |  |  |
| Lauraceae | *Litsea panamonja* | HITBC J290 | KX546963 | KX545863 |  |  |  |
| Lauraceae | *Litsea pierrei* | HITBC J227 | KX546964 | KX545864 |  |  |  |
| Lauraceae | *Litsea rotundifolia* | HITBC CXQ0047 | KX546965 | KX545865 | KX546212 | KX546464 | KX546659 |
| Lauraceae | *Litsea rotundifolia var. oblongifolia* | HITBC CXQ0008 | KX546966 | KX545866 | KX546213 |  | KX546660 |
| Lauraceae | *Litsea rotundifolia var. oblongifolia* | HITBC CXQ0311 | KX546967 | KX545867 |  |  |  |
| Lauraceae | *Litsea rotundifolia var.oblongifolia* | HITBC GBOW0315 | KX546968 | KX545868 | KX546214 |  | KX546661 |
| Lauraceae | *Litsea rotundifolia var.oblongifolia* | HITBC CXQ0009 | KX546969 | KX545869 | KX546215 |  | KX546662 |
| Lauraceae | *Litsea rotundifolia var.oblongifolia* | HITBC CXQ0026 | KX546970 | KX545870 | KX546216 |  | KX546663 |
| Lauraceae | *Litsea rubescens* | HITBC CXQ0414 | KX546971 | KX545871 | KX546217 | KX546465 | KX546664 |
| Lauraceae | *Litsea rubescens* | HITBC CXQ0422 | KX546972 | KX545872 | KX546218 | KX546466 | KX546665 |
| Lauraceae | *Litsea rubescens* | HITBC CXQ09053 | KX546973 | KX545873 | KX546219 |  | KX546666 |
| Lauraceae | *Litsea salicifolia* | HITBC CXQ0103 | KX546974 | KX545874 | KX546220 |  | KX546667 |
| Lauraceae | *Litsea salicifolia* | HITBC CXQ0110 | KX546975 | KX545875 | KX546221 |  |  |
| Lauraceae | *Litsea salicifolia* | HITBC G198 | KX546976 | KX545876 | KX546222 |  |  |
| Lauraceae | *Litsea salicifolia* | HITBC J130 | KX546977 | KX545877 | KX546223 |  |  |
| Lauraceae | *Litsea szemois* | HITBC J162 | KX546978 | KX545878 |  |  |  |
| Lauraceae | *Litsea szemois* | HITBC J256 | KX546979 | KX545879 |  |  |  |
| Lauraceae | *Litsea szemois* | HITBC J263 | KX546980 |  | KX546224 |  |  |
| Lauraceae | *Litsea szemois* | HITBC J672 | KX546981 | KX545880 |  |  |  |
| Lauraceae | *Litsea verticillata* | HITBC J388 | KX546982 | KX545881 |  |  |  |
| Lauraceae | *Litsea wilsonii* | HITBC CXQ0435 | KX546983 | KX545882 | KX546225 | KX546467 | KX546668 |
| Lauraceae | *Litsea wilsonii* | HITBC CXQ0446 | KX546984 | KX545883 | KX546226 | KX546468 | KX546669 |
| Lauraceae | *Litsea wilsonii* | HITBC CXQ0458 | KX546985 | KX545884 | KX546227 |  | KX546670 |
| Lauraceae | *Litsea yunnanensis* | HITBC Q137 | KX546986 | KX545885 | KX546228 | KX546469 | KX546671 |
| Lauraceae | *Machilus breviflora* | HITBC LJ2006013 | KX546987 | KX545886 | KX546229 | KX546470 | KX546672 |
| Lauraceae | *Machilus breviflora* | HITBC LJ2006043 | KX546988 | KX545887 | KX546230 | KX546471 | KX546673 |
| Lauraceae | *Machilus breviflora* | HITBC LJ2006045 | KX546989 | KX545888 | KX546231 | KX546472 | KX546674 |
| Lauraceae | *Machilus chienkweiensis* | HITBC LJ2002153 | KX546990 | KX545889 | KX546232 | KX546473 | KX546675 |
| Lauraceae | *Machilus chinensis* | HITBC LJ2006090 | KX546991 | KX545890 | KX546233 | KX546474 | KX546676 |
| Lauraceae | *Machilus chinensis* | HITBC LJ2006091 | KX546992 | KX545891 | KX546234 |  |  |
| Lauraceae | *Machilus chinensis* | HITBC LJ2009004 | KX546993 | KX545892 | KX546235 | KX546475 | KX546677 |
| Lauraceae | *Machilus chuanchienensis* | HITBC CXQ0419 | KX546994 | KX545893 | KX546236 |  | KX546678 |
| Lauraceae | *Machilus chuanchienensis* | HITBC CXQ0423 | KX546995 | KX545894 | KX546237 |  | KX546679 |
| Lauraceae | *Machilus chuanchienensis* | HITBC CXQ0434 | KX546996 | KX545895 | KX546238 |  | KX546680 |
| Lauraceae | *Machilus decursinervis* | HITBC CXQ0010 | KX546997 | KX545896 | KX546239 | KX546476 | KX546681 |
| Lauraceae | *Machilus decursinervis* | HITBC CXQ0023 | KX546998 | KX545897 | KX546240 |  | KX546682 |
| Lauraceae | *Machilus decursinervis* | HITBC CXQ0039 | KX546999 | KX545898 | KX546241 | KX546477 | KX546683 |
| Lauraceae | *Machilus duthiei* | HITBC LJ2006094 | KX547000 | KX545899 | KX546242 | KX546478 | KX546684 |
| Lauraceae | *Machilus duthiei* | HITBC LJ603 | KX547001 | KX545900 | KX546243 |  |  |
| Lauraceae | *Machilus fasciculata* | HITBC GBOW0040 | KX547002 | KX545901 | KX546244 |  |  |
| Lauraceae | *Machilus fasciculata* | HITBC GBOW0274 |  | KX545902 | KX546245 |  | KX546685 |
| Lauraceae | *Machilus gamblei* | HITBC CXQ764 | KX547003 | KX545903 | KX546246 | KX546479 | KX546686 |
| Lauraceae | *Machilus gamblei* | HITBC CXQ816 | KX547004 | KX545904 | KX546247 |  |  |
| Lauraceae | *Machilus gamblei* | HITBC CXQ854 |  | KX545905 | KX546248 |  |  |
| Lauraceae | *Machilus gamblei* | HITBC LJ2002084 | KX547005 | KX545906 | KX546249 |  |  |
| Lauraceae | *Machilus gamblei* | HITBC LJ2002089 | KX547006 | KX545907 | KX546250 | KX546480 | KX546687 |
| Lauraceae | *Machilus gamblei* | HITBC LJ2002143 | KX547007 | KX545908 |  |  |  |
| Lauraceae | *Machilus gamblei* | HITBC LJ2002148 | KX547008 | KX545909 |  |  | KX546688 |
| Lauraceae | *Machilus gamblei* | HITBC LJ2006015 | KX547009 | KX545910 | KX546251 | KX546481 | KX546689 |
| Lauraceae | *Machilus gamblei* | HITBC LJ2006044 | KX547010 | KX545911 | KX546252 | KX546482 | KX546690 |
| Lauraceae | *Machilus grijsii* | HITBC Q109 | KX547011 | KX545912 | KX546253 |  |  |
| Lauraceae | *Machilus grijsii* | HITBC GBOW0930 | KX547012 | KX545913 | KX546254 |  | KX546691 |
| Lauraceae | *Machilus grijsii* | HITBC GBOW0936 | KX547013 | KX545914 | KX546255 | KX546483 | KX546615 |
| Lauraceae | *Machilus grijsii* | HITBC LJ2006028 | KX547014 | KX545915 | KX546256 | KX546484 | KX546692 |
| Lauraceae | *Machilus grijsii* | HITBC LJ2006033 | KX547015 | KX545916 | KX546257 | KX546485 | KX546693 |
| Lauraceae | *Machilus grijsii* | HITBC LJ2006042 | KX547016 | KX545917 | KX546258 | KX546486 | KX546694 |
| Lauraceae | *Machilus grijsii* | HITBC LJ2007211 |  | KX545918 | KX546259 |  |  |
| Lauraceae | *Machilus ichangensis* | HITBC CXQ0430 | KX547017 | KX545919 | KX546260 | KX546487 | KX546695 |
| Lauraceae | *Machilus ichangensis* | HITBC CXQ0432 | KX547018 | KX545920 | KX546261 | KX546488 | KX546696 |
| Lauraceae | *Machilus ichangensis* | HITBC CXQ0433 | KX547019 | KX545921 | KX546262 | KX546489 | KX546697 |
| Lauraceae | *Machilus kurzii* | HITBC CXQ0005 | KX547020 | KX545922 | KX546263 | KX546490 | KX546698 |
| Lauraceae | *Machilus kurzii* | HITBC CXQ0024 | KX547021 | KX545923 | KX546264 |  | KX546699 |
| Lauraceae | *Machilus kurzii* | HITBC CXQ0107 | KX547022 | KX545924 | KX546265 |  | KX546700 |
| Lauraceae | *Machilus kwangtungensis* | HITBC LJ2006019 | KX547023 | KX545925 | KX546266 | KX546491 | KX546701 |
| Lauraceae | *Machilus kwangtungensis* | HITBC LJ2006027 | KX547024 | KX545926 | KX546267 | KX546492 | KX546702 |
| Lauraceae | *Machilus kwangtungensis* | HITBC LJ2006032 | KX547025 | KX545927 | KX546268 | KX546493 | KX546703 |
| Lauraceae | *Machilus leptophylla* | HITBC LJ2007190 |  | KX545928 | KX546269 |  |  |
| Lauraceae | *Machilus leptophylla* | HITBC LJ2007228 | KX547026 | KX545929 | KX546270 |  |  |
| Lauraceae | *Machilus longipedicellata* | HITBC CXQ578 | KX547027 | KX545930 | KX546271 |  |  |
| Lauraceae | *Machilus longipedicellata* | HITBC CXQ638 | KX547028 | KX545931 | KX546272 |  |  |
| Lauraceae | *Machilus longipedicellata* | HITBC CXQ701 | KX547029 | KX545932 | KX546273 |  |  |
| Lauraceae | *Machilus longipedicellata* | HITBC CXQ724 | KX547030 | KX545933 | KX546274 |  |  |
| Lauraceae | *Machilus minutiflora* | HITBC GBOW0206 | KX547031 | KX545934 | KX546275 |  | KX546773 |
| Lauraceae | *Machilus minutiflora* | HITBC GBOW1428 |  | KX545935 |  |  | KX546774 |
| Lauraceae | *Machilus nanmu* | HITBC J401 | KX547032 | KX545936 |  |  |  |
| Lauraceae | *Machilus nanmu* | HITBC LJ2007263 | KX547033 | KX545937 | KX546276 |  |  |
| Lauraceae | *Machilus nanmu* | HITBC LJ2007267 | KX547034 | KX545938 | KX546277 |  |  |
| Lauraceae | *Machilus oreophila* | HITBC LJ2002176 |  | KX545939 | KX546278 | KX546494 | KX546704 |
| Lauraceae | *Machilus oreophila* | HITBC LJ2002211 | KX547035 | KX545940 | KX546279 | KX546495 | KX546705 |
| Lauraceae | *Machilus oreophila* | HITBC LJ2002212 | KX547036 | KX545941 | KX546280 | KX546496 | KX546706 |
| Lauraceae | *Machilus oreophila* | HITBC LJ2005012 | KX547037 | KX545942 | KX546281 | KX546497 | KX546707 |
| Lauraceae | *Machilus oreophila* | HITBC LJ2006067 | KX547038 | KX545943 | KX546282 | KX546498 | KX546708 |
| Lauraceae | *Machilus pauhoi* | HITBC CXQ0259 |  |  | KX546283 |  |  |
| Lauraceae | *Machilus pauhoi* | HITBC CXQ0407 | KX547039 | KX545944 | KX546284 |  | KX546709 |
| Lauraceae | *Machilus platycarpa* | HITBC LJ2006073 | KX547040 | KX545945 |  |  |  |
| Lauraceae | *Machilus platycarpa* | HITBC LJ2006087 | KX547041 | KX545946 |  |  |  |
| Lauraceae | *Machilus pomifera* | HITBC LJ2006064 | KX547042 | KX545947 | KX546285 | KX546499 | KX546710 |
| Lauraceae | *Machilus pomifera* | HITBC LJ2006079 | KX547043 | KX545948 |  | KX546500 | KX546711 |
| Lauraceae | *Machilus pomifera* | HITBC LJ2006081 | KX547044 | KX545949 | KX546286 | KX546501 | KX546712 |
| Lauraceae | *Machilus robusta* | HITBC CXQ1017 | KX547045 | KX545950 | KX546287 |  |  |
| Lauraceae | *Machilus robusta* | HITBC GBOW0246 | KX547046 | KX545951 |  |  |  |
| Lauraceae | *Machilus robusta* | HITBC LJ2002086 | KX547047 | KX545952 | KX546288 | KX546502 | KX546713 |
| Lauraceae | *Machilus robusta* | HITBC LJ2002116 | KX547048 | KX545953 | KX546289 | KX546503 | KX546714 |
| Lauraceae | *Machilus robusta* | HITBC LJ2002145 | KX547049 | KX545954 | KX546290 |  |  |
| Lauraceae | *Machilus robusta* | HITBC Q118 | KX547050 | KX545955 | KX546291 |  |  |
| Lauraceae | *Machilus rufipes* | HITBC CXQ898 | KX547051 | KX545956 | KX546292 |  |  |
| Lauraceae | *Machilus rufipes* | HITBC CXQ906 | KX547052 | KX545957 | KX546293 |  |  |
| Lauraceae | *Machilus rufipes* | HITBC J070 | KX547053 |  |  |  |  |
| Lauraceae | *Machilus rufipes* | HITBC Q081 | KX547054 |  | KX546294 |  |  |
| Lauraceae | *Machilus rufipes* | HITBC Q110 | KX547055 | KX545958 | KX546295 |  |  |
| Lauraceae | *Machilus salicina* | HITBC LJ2002040 | KX547056 | KX545959 | KX546296 | KX546504 | KX546715 |
| Lauraceae | *Machilus salicina* | HITBC LJ2002103 | KX547057 | KX545960 | KX546297 | KX546505 | KX546716 |
| Lauraceae | *Machilus salicina* | HITBC LJ2002105 | KX547058 | KX545961 | KX546298 | KX546506 | KX546717 |
| Lauraceae | *Machilus salicina* | HITBC LJ2006038 | KX547059 | KX545962 |  | KX546507 | KX546718 |
| Lauraceae | *Machilus salicina* | HITBC LJ2007220 |  | KX545963 | KX546299 |  |  |
| Lauraceae | *Machilus salicina* | HITBC LJ2009013 | KX547060 | KX545964 | KX546300 |  |  |
| Lauraceae | *Machilus shweliensis* | HITBC LJ2002055 | KX547061 | KX545965 | KX546301 | KX546508 | KX546719 |
| Lauraceae | *Machilus shweliensis* | HITBC LJ2002065 | KX547062 | KX545966 | KX546302 | KX546509 | KX546720 |
| Lauraceae | *Machilus shweliensis* | HITBC LJ2002085 | KX547063 | KX545967 | KX546303 | KX546510 | KX546721 |
| Lauraceae | *Machilus sichuanensis* | HITBC LJ2002087 | KX547064 | KX545968 | KX546304 | KX546511 | KX546722 |
| Lauraceae | *Machilus sichuanensis* | HITBC CXQ0460 | KX547065 | KX545969 | KX546305 |  |  |
| Lauraceae | *Machilus sichuanensis* | HITBC CXQ0462 | KX547066 | KX545970 | KX546306 |  | KX546723 |
| Lauraceae | *Machilus sichuanensis* | HITBC CXQ0465 | KX547067 | KX545971 | KX546307 |  | KX546724 |
| Lauraceae | *Machilus tenuipilis* | HITBC GBOW1092 | KX547068 | KX545972 | KX546308 | KX546512 | KX546725 |
| Lauraceae | *Machilus tenuipilis* | HITBC LJ2002035 | KX547069 | KX545973 | KX546309 | KX546513 | KX546726 |
| Lauraceae | *Machilus tenuipilis* | HITBC LJ2002038 | KX547070 | KX545974 | KX546310 | KX546514 | KX546727 |
| Lauraceae | *Machilus thunbergii* | HITBC CXQ0278 | KX547071 | KX545975 | KX546311 | KX546515 | KX546728 |
| Lauraceae | *Machilus thunbergii* | HITBC CXQ0305 | KX547072 | KX545976 | KX546312 |  |  |
| Lauraceae | *Machilus thunbergii* | HITBC CXQ0406 | KX547073 | KX545977 | KX546313 |  | KX546729 |
| Lauraceae | *Machilus thunbergii* | HITBC CXQ0409 | KX547074 | KX545978 | KX546314 |  | KX546730 |
| Lauraceae | *Machilus velutina* | HITBC CXQ0055 | KX547075 | KX545979 | KX546315 |  | KX546731 |
| Lauraceae | *Machilus velutina* | HITBC CXQ0397 | KX547076 | KX545980 | KX546316 | KX546516 | KX546732 |
| Lauraceae | *Machilus velutina* | HITBC CXQ0408 | KX547077 | KX545981 | KX546317 |  | KX546733 |
| Lauraceae | *Machilus yunnanensis* | HITBC CXQ1250 | KX547078 | KX545982 | KX546318 |  |  |
| Lauraceae | *Machilus yunnanensis* | HITBC CXQ1262 | KX547079 | KX545983 | KX546319 |  |  |
| Lauraceae | *Machilus yunnanensis* | HITBC CXQ1276 | KX547080 | KX545984 | KX546320 |  |  |
| Lauraceae | *Machilus yunnanensis* | HITBC GBOW0678 | KX547081 | KX545985 |  |  |  |
| Lauraceae | *Neocinnamomum delavayi* | HITBC CXQ1001 | KX547082 | KX545986 | KX546321 | KX546517 | KX546734 |
| Lauraceae | *Neocinnamomum delavayi* | HITBC CXQ1015 | KX547083 | KX545987 | KX546322 | KX546518 | KX546735 |
| Lauraceae | *Neocinnamomum delavayi* | HITBC CXQ1018 | KX547084 | KX545988 | KX546323 | KX546519 | KX546736 |
| Lauraceae | *Neocinnamomum delavayi* | HITBC CXQ1028 | KX547085 | KX545989 | KX546324 | KX546520 | KX546737 |
| Lauraceae | *Neolitsea aurata var. aurata* | HITBC LJ2002181 | KX547086 | KX545990 | KX546325 | KX546521 | KX546738 |
| Lauraceae | *Neolitsea aurata var. aurata* | HITBC LJ2002182 | KX547087 | KX545991 | KX546326 | KX546522 | KX546739 |
| Lauraceae | *Neolitsea aurata var.chekiangensis* | HITBC LJ2007197 |  | KX545992 | KX546327 |  |  |
| Lauraceae | *Neolitsea aurata var. chekiangensis* | HITBC LJ2007229 | KX547088 | KX545993 | KX546328 |  |  |
| Lauraceae | *Neolitsea aurata var. paraciculata* | HITBC CXQ0253 | KX547089 | KX545994 | KX546329 | KX546523 | KX546740 |
| Lauraceae | *Neolitsea aurata var. paraciculata* | HITBC CXQ0296 | KX547090 | KX545995 | KX546330 |  |  |
| Lauraceae | *Neolitsea aurata var. paraciculata* | HITBC CXQ0332 | KX547091 | KX545996 | KX546331 |  | KX546741 |
| Lauraceae | *Neolitsea cambodiana var. cambodiana* | HITBC LJ2006008 | KX547092 | KX545997 |  |  |  |
| Lauraceae | *Neolitsea cambodiana var. cambodiana* | HITBC LJ2006046 | KX547093 | KX545998 |  |  |  |
| Lauraceae | *Neolitsea cambodiana var. cambodiana* | HITBC LJ2006058 | KX547094 | KX545999 |  |  |  |
| Lauraceae | *Neolitsea cambodiana var. cambodiana* | HITBC LJ2007372 | KX547095 | KX546000 |  |  |  |
| Lauraceae | *Neolitsea chuii* | HITBC CXQ644 | KX547096 | KX546001 | KX546332 |  |  |
| Lauraceae | *Neolitsea chuii* | HITBC CXQ730 | KX547097 | KX546002 | KX546333 |  |  |
| Lauraceae | *Neolitsea chuii* | HITBC LJ2002063 | KX547098 | KX546003 | KX546334 | KX546524 | KX546742 |
| Lauraceae | *Neolitsea chuii* | HITBC LJ2002068 |  | KX546004 |  | KX546525 | KX546743 |
| Lauraceae | *Neolitsea chuii* | HITBC LJ2002102 | KX547099 | KX546005 |  | KX546526 | KX546744 |
| Lauraceae | *Neolitsea chuii* | HITBC LJ2002157 | KX547100 | KX546006 | KX546335 | KX546527 | KX546745 |
| Lauraceae | *Neolitsea chuii* | HITBC LJ2002188 | KX547101 | KX546007 | KX546336 |  |  |
| Lauraceae | *Neolitsea chuii* | HITBC LJ2002196 | KX547102 | KX546008 | KX546337 |  |  |
| Lauraceae | *Neolitsea chuii* | HITBC LJ2006083 | KX547103 |  | KX546338 | KX546528 | KX546746 |
| Lauraceae | *Neolitsea chuii* | HITBC Q079 | KX547104 | KX546009 | KX546339 |  |  |
| Lauraceae | *Neolitsea chuii* | HITBC Q080 | KX547105 | KX546010 | KX546340 |  |  |
| Lauraceae | *Neolitsea confertifolia* | HITBC LJ2007289 | KX547106 | KX546011 | KX546341 | KX546529 | KX546747 |
| Lauraceae | *Neolitsea confertifolia* | HITBC LJ2007290 | KX547107 | KX546012 | KX546342 | KX546530 | KX546748 |
| Lauraceae | *Neolitsea confertifolia* | HITBC LJ2007319 | KX547108 | KX546013 | KX546343 |  |  |
| Lauraceae | *Neolitsea homilantha* | HITBC CXQ546 | KX547109 | KX546014 | KX546344 |  |  |
| Lauraceae | *Neolitsea homilantha* | HITBC CXQ768 | KX547110 | KX546015 | KX546345 |  |  |
| Lauraceae | *Neolitsea homilantha* | HITBC GBOW0237 | KX547111 | KX546016 | KX546346 | KX546531 | KX546749 |
| Lauraceae | *Neolitsea homilantha* | HITBC LJ0013 | KX547112 | KX546017 | KX546347 |  |  |
| Lauraceae | *Neolitsea homilantha* | HITBC LJ2002058 | KX547113 | KX546018 |  |  |  |
| Lauraceae | *Neolitsea homilantha* | HITBC LJ2002071 | KX547114 | KX546019 | KX546348 | KX546532 | KX546750 |
| Lauraceae | *Neolitsea homilantha* | HITBC Q083 | KX547115 | KX546020 | KX546349 | KX546533 | KX546751 |
| Lauraceae | *Neolitsea homilantha* | HITBC Q125 | KX547116 | KX546021 | KX546350 | KX546534 | KX546752 |
| Lauraceae | *Neolitsea levinei* | HITBC GBOW0679 | KX547117 | KX546023 | KX546351 | KX546535 | KX546753 |
| Lauraceae | *Neolitsea levinei* | HITBC LJ2002003 | KX547118 | KX546024 | KX546352 |  |  |
| Lauraceae | *Neolitsea levinei* | HITBC LJ2002180 | KX547119 | KX546022 |  | KX546536 | KX546754 |
| Lauraceae | *Neolitsea levinei* | HITBC LJ2007363 | KX547120 | KX546025 | KX546353 | KX546537 | KX546755 |
| Lauraceae | *Neolitsea levinei* | HITBC LJ2009108 | KX547121 | KX546026 | KX546354 | KX546538 | KX546756 |
| Lauraceae | *Neolitsea phanerophlebia* | HITBC LJ2006068 | KX547122 | KX546027 | KX546355 | KX546539 | KX546757 |
| Lauraceae | *Neolitsea phanerophlebia* | HITBC LJ2006083 |  | KX546028 |  |  |  |
| Lauraceae | *Neolitsea pinninervis* | HITBC LJ2002183 | KX547123 | KX546029 | KX546356 | KX546540 | KX546758 |
| Lauraceae | *Neolitsea pinninervis* | HITBC LJ2002187 | KX547124 | KX546030 | KX546357 | KX546541 | KX546759 |
| Lauraceae | *Neolitsea pinninervis* | HITBC LJ2002197 | KX547125 | KX546031 | KX546358 | KX546542 | KX546760 |
| Lauraceae | *Neolitsea polycarpa* | HITBC CXQ738 | KX547126 | KX546032 | KX546359 |  |  |
| Lauraceae | *Neolitsea pulchella* | HITBC LJ2002141 | KX547127 | KX546033 |  |  |  |
| Lauraceae | *Neolitsea pulchella* | HITBC LJ2002146 |  |  |  | KX546543 | KX546761 |
| Lauraceae | *Neolitsea pulchella* | HITBC LJ2002166 | KX547128 | KX546034 | KX546360 |  |  |
| Lauraceae | *Neolitsea sericea* | HITBC CXQ0315 | KX547129 | KX546035 | KX546361 |  |  |
| Lauraceae | *Neolitsea sericea* | HITBC CXQ0316 | KX547130 | KX546036 | KX546362 |  |  |
| Lauraceae | *Neolitsea sericea* | HITBC CXQ0317 | KX547131 | KX546037 | KX546363 |  |  |
| Lauraceae | *Neolitsea shingningensis* | HITBC CXQ0282 | KX547132 | KX546038 | KX546364 |  | KX546762 |
| Lauraceae | *Neolitsea shingningensis* | HITBC CXQ0286 | KX547133 | KX546039 | KX546365 |  | KX546763 |
| Lauraceae | *Neolitsea sutchuanensis* | HITBC LJ2002101 | KX547134 | KX546040 | KX546366 | KX546544 | KX546764 |
| Lauraceae | *Neolitsea sutchuanensis* | HITBC LJ2007261 | KX547135 | KX546041 | KX546367 | KX546545 | KX546765 |
| Lauraceae | *Neolitsea velutina* | HITBC CXQ0472 |  | KX546042 |  |  |  |
| Lauraceae | *Neolitsea velutina* | HITBC CXQ0474 | KX547136 | KX546043 | KX546368 | KX546546 | KX546766 |
| Lauraceae | *Neolitsea velutina* | HITBC CXQ0475 | KX547137 | KX546044 | KX546369 | KX546547 | KX546767 |
| Lauraceae | *Neolitsea velutina* | HITBC CXQ0478 | KX547138 | KX546045 | KX546370 |  | KX546768 |
| Lauraceae | *Neolitsea zeylanica* | HITBC CXQ0025 | KX547139 | KX546046 | KX546371 |  | KX546769 |
| Lauraceae | *Neolitsea zeylanica* | HITBC CXQ0072 | KX547140 | KX546047 | KX546372 |  |  |
| Lauraceae | *Phoebe chekiangensis* | HITBC CXQ0363 | KX547141 | KX546048 | KX546373 |  |  |
| Lauraceae | *Phoebe chekiangensis* | HITBC CXQ0376 | KX547142 | KX546049 | KX546374 |  |  |
| Lauraceae | *Phoebe chekiangensis* | HITBC CXQ0412 | KX547143 | KX546050 | KX546375 |  |  |
| Lauraceae | *Phoebe glaucifolia* | HITBC CXQ1266 | KX547144 | KX546051 | KX546376 |  |  |
| Lauraceae | *Phoebe glaucifolia* | HITBC CXQ1273 | KX547145 | KX546052 | KX546377 | KX546548 | KX546770 |
| Lauraceae | *Phoebe lanceolata* | HITBC G156 | KX547146 | KX546053 | KX546378 |  |  |
| Lauraceae | *Phoebe lanceolata* | HITBC G161 | KX547147 | KX546054 | KX546379 |  |  |
| Lauraceae | *Phoebe lanceolata* | HITBC J274 | KX547148 | KX546055 |  |  | KX546771 |
| Lauraceae | *Phoebe macrocarpa* | HITBC CXQ09097 | KX547149 | KX546056 | KX546380 |  | KX546772 |
| Lauraceae | *Phoebe macrocarpa* | HITBC GBOW0160 | KX547150 | KX546057 | KX546381 |  |  |
| Lauraceae | *Machilus minutiflora* | HITBC GBOW0699 | KX547151 | KX546058 | KX546382 |  |  |
| Lauraceae | *Phoebe neurantha* | HITBC CXQ0208 | KX547152 | KX546059 | KX546383 | KX546549 | KX546775 |
| Lauraceae | *Phoebe neurantha* | HITBC CXQ0453 | KX547153 | KX546060 | KX546384 | KX546550 | KX546776 |
| Lauraceae | *Phoebe neurantha* | HITBC CXQ09023 | KX547154 | KX546061 | KX546385 |  | KX546777 |
| Lauraceae | *Phoebe neurantha* | HITBC GBOW1318 | KX547155 | KX546062 | KX546386 |  |  |
| Lauraceae | *Phoebe puwenensis* | HITBC CXQ0104 | KX547156 | KX546063 | KX546387 |  | KX546778 |
| Lauraceae | *Phoebe puwenensis* | HITBC CXQ0109 | KX547157 | KX546064 | KX546388 | KX546551 | KX546779 |
| Lauraceae | *Phoebe puwenensis* | HITBC J337 |  | KX546065 |  |  |  |
| Lauraceae | *Phoebe puwenensis* | HITBC J589 | KX547158 | KX546066 |  |  |  |
| Lauraceae | *Phoebe rufescens* | HITBC Q143 | KX547159 |  | KX546389 |  |  |
| Lauraceae | *Phoebe sheareri* | HITBC CXQ0213 | KX547160 | KX546067 | KX546390 | KX546552 | KX546780 |
| Lauraceae | *Phoebe sheareri* | HITBC CXQ0308 | KX547161 | KX546068 | KX546391 |  |  |
| Lauraceae | *Phoebe sheareri* | HITBC CXQ0365 | KX547162 | KX546069 | KX546392 | KX546553 | KX546781 |
| Lauraceae | *Phoebe tavoyana* | HITBC CXQ0426 | KX547163 | KX546070 | KX546393 | KX546554 | KX546782 |
