# Supplementary figures and images for: DNA barcoding evaluation and implications for phylogenetic relationships in Lauraceae from China
Source: PLoS One. 2017 Apr 17;12(4):e0175788. doi: 10.1371/journal.pone.0175788 (PMC5393608; doi:10.1371/journal.pone.0175788)

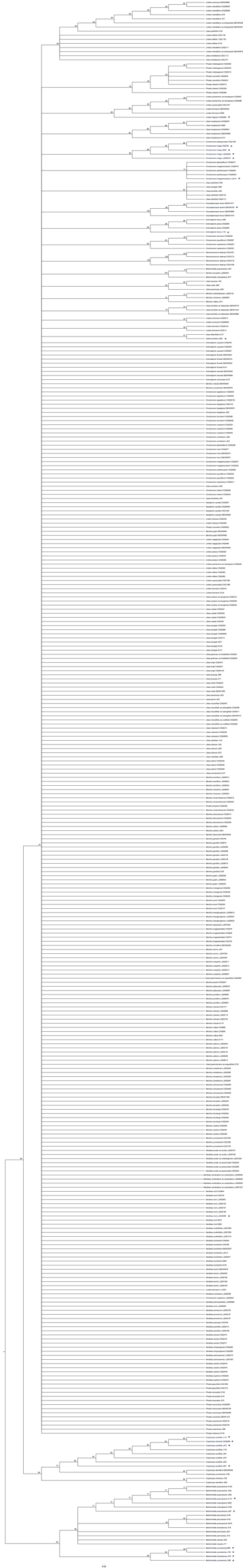

Supplement: S1 Fig — Erroneous identifications which recognized based on rbcL marker at the genus and species levels are marked by stars. (TIFF) [file pone.0175788.s004.tiff]

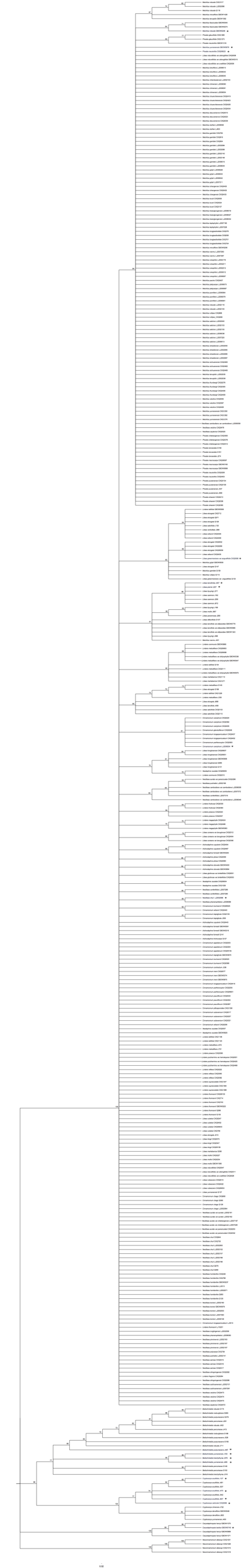

Supplement: S2 Fig — Erroneous identifications which recognized based on matK marker at the genus and species levels are marked by stars. (TIFF) [file pone.0175788.s005.tiff]
